# Cuproptosis as a novel predictor of immunotherapy response and shapes the immune landscape in pan-cancer analysis

**DOI:** 10.1007/s12672-026-05070-5

**Published:** 2026-05-01

**Authors:** Yitong Pan, Xiaodi Hu, Jun Cheng

**Affiliations:** 1 Furong Laboratory, Central South University, Changsha, 410078 Hunan China; 2 Hunan Cancer Hospital and The Affiliated Cancer Hospital of Xiangya School of Medicine, Central South University, Changsha, 410013 Hunan China

**Keywords:** Cuproptosis, Regulated cell death, Antitumor immunity, Multi-omics immune landscapes, Immunotherapy

## Abstract

The heterogeneity of cancer renders its response to immunotherapy elusive, warranting the identification of robust biomarkers for evaluation. As a form of recently defined regulated cell death (RCD), cuproptosis is driven by copper-dependent proteotoxic stress in mitochondria tricarboxylic acid cycle (TCA cycle), exerting a sophisticated role in antitumor immunity, yet remains poorly understood. Here, we established a cuproptosis score to characterize the immune landscape across 23 human cancers and established the correlation of cuproptosis score with patient immunotherapy outcomes through multi-omics analysis. Bulk and single-cell transcriptomic analysis revealed that with cuproptosis-low patients tend to have improved immunotherapy outcomes compared to those with high cuproptosis scores, with increased immune infiltration and function, as well as abundant cytokines, checkpoints and major histocompatibility complex (MHC) molecules expression. Spatial transcriptomic analysis indicated that immune cells exhibited higher cuproptosis scores than tumor cells, hinting that immune cells are vulnerable to cuproptosis. Further metabolomic and transcriptomic analysis of underlying intrinsic features revealed that cuproptosis-low tumors exhibited a metabolic and signaling pathway profile enrichment favoring antitumor immunity and potent immunogenicity. Overall, our multi-omics analysis suggests cuproptosis score as a robust biomarker for immunotherapy benefit in multiple cancers, reveals cuproptosis-related extrinsic and intrinsic immune landscapes, and provides a broad framework for understanding the relevance of cuproptosis to cancer immunology and clinical benefits of immunotherapy.

**Supplementary Information:**

The online version contains supplementary material available at 10.1007/s12672-026-05070-5.

## Introduction

Hitherto, the continuous advances in the immuno-oncology and the development of immunotherapeutic agents, such as checkpoint inhibitors (CPIs) and chimeric antigen receptor (CAR) T cell therapy, provide promising strategies to overcome tumors by activating endogenous immune system [1]. However, there remain many patients with limited or absent benefit from immunotherapy. Therefore, identifying the population eligible for immunotherapy is a burning challenge at this stage.

Multi-modulators anchor the efficacy of immunotherapy in a sophisticated network. The tumor immune microenvironment (TME) is one of the determinants of immunotherapy efficacy, in which the proportion of infiltrating immune cells and the composition of immune cells contribute significantly to antitumor immunity [2–4]. Equally, the fraction of various cytokines, the expression of immune checkpoints, and MHC molecule-mediated antigen presentation are critical to the immunotherapeutic response [1]. Nevertheless, TME varies extensively among cancers and even individuals, presumably related to the tumor genetic heterogeneity [5, 6]. On top of that, previous studies have proposed several biomarkers linked to the immune response, such as tumor mutation burden (TMB), essential to initiate the cancer-immunity cycle [7]. In addition, immune-related signaling and metabolic reprogramming in the TME manipulate antitumor immunity, with aggressive oxidative phosphorylation (OXPHOS) and fatty acid metabolism impairing the survival and function of immune effector cells [7, 8]. However, given the complex interactions between the tumors and the immune system, such biomarkers cannot fully stratify the patients for optimal benefit. For instance, the commonly used TMB could be ineffective in predicting benefit from the combination of anti-Programmed Cell Death 1 (anti-PD-1) and anti-Cytotoxic T-Lymphocyte Associated Protein 4 (anti-CTLA-4) in several cancers [7], highlighting the necessity to establish robust markers and optimize biomarker combinations.

Regulatory cell death (RCD) is not only recognized as a natural obstacle to tumorigenesis and progression, but also exerts an important and intricate role in TME and immunotherapy [9–11]. As the most recently identified form of RCD, cuproptosis is triggered by copper-dependent proteotoxic stress in mitochondria TCA cycle, distinct from other types of RCD [12]. Mechanistically, copper directly binds to lipoylated proteins and causes aggregation of lipoylated proteins, resulting in a toxic gain of function in TCA cycle, where Ferredoxin 1 (FDX1), lipoic acid pathway, and pyruvate dehydrogenase (PDH) complexes function as governors in cuproptosis regulation [12]. Mitochondrial metabolism, especially the active TCA cycle, electron transport chain (ETC)/OXPHOS, and fatty acid metabolism, can increase lipoylated TCA enzymes and favor cuproptosis, whereas glycolysis restrains such process, suggesting cuproptosis as a metabolism-modulated cell death [12]. Interestingly, cuproptosis seems to play a complex role in antitumor immunity. Cellular copper accumulation can trigger immunogenic cell death and promote antitumor immunity; nevertheless, several studies suggested that copper depletion supports immunotherapy with increased tumor-infiltrating T cells and Natural Killer (NK) cells and decreased immunosuppressive cells [13–15]. Notably, cuproptosis-related TCA cycle, OXPHOS, or fatty acid metabolism is also engaged in the regulation of TME and antitumor immunity [7, 8, 16]. To date, however, the involvement of cuproptosis in TME and immunotherapy outcomes in pan-cancer remains poorly characterized.

In this study, we established a cuproptosis-related score and presented a multi-omics pan-cancer analysis to characterize cuproptosis-related immunotherapeutic outcomes and underlying immune landscapes across 23 cancers using the Cancer Genome Atlas (TCGA) datasets and the Gene Expression Omnibus (GEO) datasets. Specifically, bulk and single-cell transcriptomics revealed that patients with high cuproptosis score exhibited compromised immunotherapy benefits with impaired immune infiltration and function. Spatial transcriptomics depicted that tumor tissues are generally characterized by low cuproptosis score compared with TILs regions. Further metabolic and transcriptomic analysis suggested perturbations of immune-related metabolism and signaling pathways as potential causes for poor immunotherapy efficacy in patients with high cuproptosis score. Collectively, our comprehensive analysis provides insights into understanding the involvment of cuproptosis in immuno-oncology and immunotherapy.

## Results

### Cuproptosis score as a promising predictor for immunotherapy outcomes

**Fig. 1:**
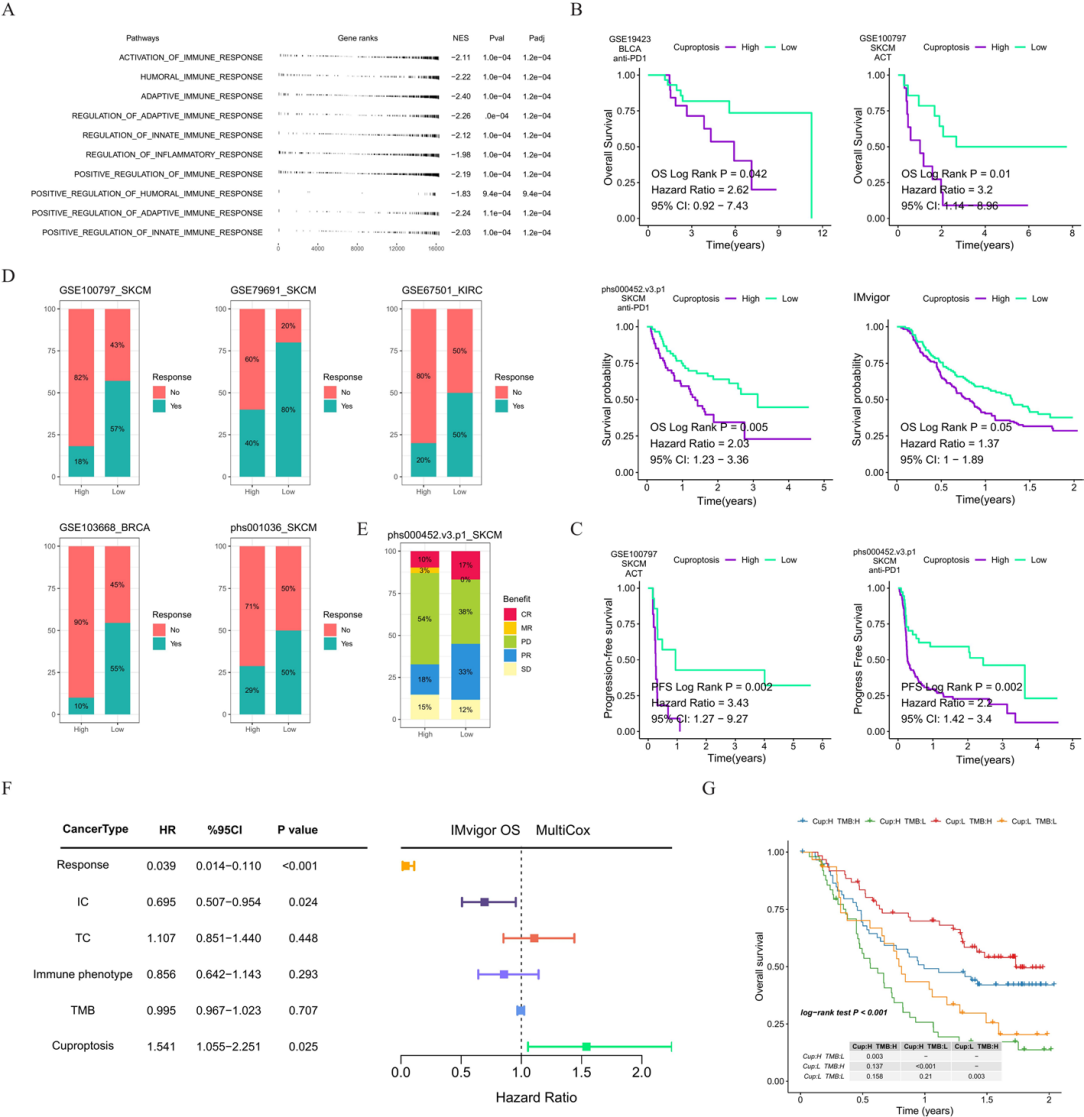
Cuproptosis score as a promising predictor for immunotherapy outcomes. **A** Enrichment of immune response-related signatures of patients with cuproptosis-high and -low groups in TCGA datasets. **B** Kaplan-Meier estimates of overall survival of patients treatmented with immunotherapy in BLCA (GSE19423, cuproptosis-high: *n* = 19; cuproptosis-low: *n* = 29), SKCM (GSE100797, cuproptosis-high: *n* = 11; cuproptosis-low: *n* = 14), SKCM (phs000452.v3.p1, cuproptosis-high: *n* = 61; cuproptosis-low: *n* = 60) and metastatic urothelial carcinoma (IMvigor210, cuproptosis-high: *n* = 133; cuproptosis-low: *n* = 107). Pval means P value and Padj means adjacent P value. **C** Kaplan-Meier estimates of progression free survival of patients treatmented with immunothreapy in SKCM datasets (GSE100797, cuproptosis-high: *n* = 11; cuproptosis-low: *n* = 14; phs000452.v3.p1, cuproptosis-high: *n* = 61; cuproptosis-low: *n* = 60). **D** The proportion of response status to immunotherapy in cuproptosis-high and -low groups in GSE100797 (cuproptosis-high: *n* = 11; cuproptosis-low: *n* = 14, P value = 0.118), GSE79691 (cuproptosis-high: *n* = 5; cuproptosis-low: *n* = 5, P value = 0.519), GSE67501 (cuproptosis-high: *n* = 5; cuproptosis-low: *n* = 6, P value = 0.689), GSE103668 (cuproptosis-high: *n* = 10; cuproptosis-low: *n* = 11, P value = 0.089), phs000452.v3.p1 (cuproptosis-high: *n* = 61; cuproptosis-low: *n* = 60, P value = 0.030). **E** The proportion of response status to immunotherapy in cuproptosis-high and -low groups in phs000452.v3.p1 (cuproptosis-high: *n* = 61; cuproptosis-low: *n* = 60, P value = 0.106). **F** Multivariate Cox regression of cuproptosis score and clinical features in metastatic urothelial carcinoma (IMvigor210). **G** Kaplan-Meier estimates of overall survival of patients with cuproptosis-high combined with TMB-high, cuproptosis-high combined with TMB-low, cuproptosis-low combined with TMB-high, and cuproptosis-low combined with TMB-low groups in metastatic urothelial carcinoma (IMvigor210). The log-rank P value differences between each two groups are shown in the table

We established a cuproptosis score based on the expression of genes directly governing copper ionophore-mediated death in the TCGA pan-cancer database and GEO datasets. Given the intricate role of copper and cuproptosis-related metabolism in antitumor immunity [13–15], we analyzed enrichment of immune response-related signatures between cuproptosis-low and -high groups by GSEA. Surprisingly, all immune response-related signatures, including humoral immune response, innate immune response, adaptive immune response, and inflammatory immune response, were significantly upregulated in cuproptosis-low group (Fig. 1A). Subsequently, we evaluated the prediction value of cuproptosis score in immunotherapy outcomes using several CPI-associated datasets. Consistently, patients with SKCM, BLCA, or metastatic urothelial carcinoma (IMvigor210) in the cuproptosis-low group had significantly improved OS or PFS after immunotherapy, including anti-PD-1, anti-PD-L1, or adoptive cell transfer therapy (e.g., CAR-T), compared to those in the cuproptosis-high group, implying that cuproptosis may compromise the benefits of immunotherapy (Fig. 1B and C). In line with this, patients with high cuproptosis scores responded poorly to CPIs treatment, whereas more than half of the patients with low cuproptosis scores responded to treatment, with a response rate of up to 80% (Fig. 1D). Specifically, high-cuproptosis group dominantly exhibited progressive disease (PD), stable disease (SD), and minimal response (MR), whereas low-cuproptosis group largely exhibited complete response (CR) and partial response (PR) (Fig. 1E).

A potential explanation is that immune cells may be extraordinarily sensitive to cuproptosis, and thus both immune cells and cancer cells suffer from cuproptosis simultaneously, thereby compromising antitumor immunity. Another possibility is that cancer cells dying from cuproptosis might impede antigen-presenting cell-mediated anti-tumor immunity. These speculations have been demonstrated in other types of RCD (see discussion) [17–19]. We further analyzed the clinicopathological data in the IMvigor210 dataset, and of note, following the removal of samples with incomplete clinical information, cuproptosis score remained an independent predictor based on multivariate Cox regression analysis, even more significant than PD-L1 expression in tumor cells (TC), immune phenotype, or tumor mutation burden (TMB) (Fig. 1F). To further extend the clinical utility of our model, we sought to combine the cuproptosis score with commonly used immunotherapy response markers. TMB is a well-established predictor for immunotherapy efficacy, and our results uncovered excellent stratification performance for cuproptosis score + TMB. As shown in Fig. 1G, patients with low cuproptosis scores and high TMB exhibited optimal immunotherapy outcomes, whereas patients with high cuproptosis scores and low TMB presented poorest immunotherapy benefits. Overall, our results suggest an unexpected predicting potential of cuproptosis score for immunotherapy outcomes and substantiate its robustness in independently predicting prognosis of immunotherapy. In addition, the combination of cuproptosis score and TMB can reinforce the potential to recognize the population with truly transformative benefits from CPIs.

### Extrinsic immune landscapes: immune function in cuproptosis-low and -high groups

**Fig. 2:**
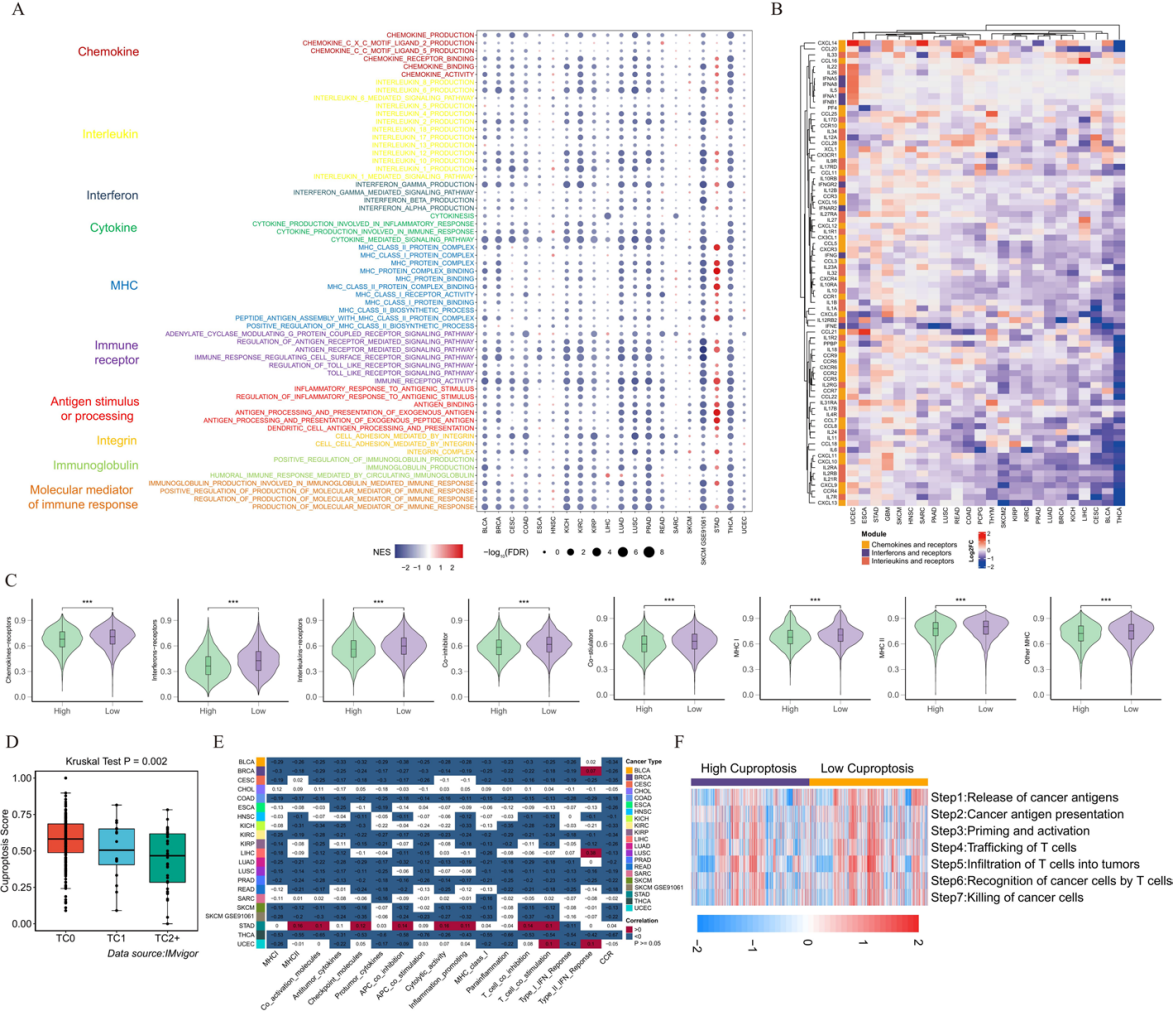
Immune function-associated features in cuproptosis-low and -high groups. **A** Enrichment of immune function-related GO pathways in patients in cuproptosis-high and -low groups from TCGA datasets and GSE91061. **B** The fold differences of immune function-associated gene expression in patients from cuproptosis-high and -low groups. Log_2_(fold change) (Log_2_FC) values are shown. **C** The violin plot of the expression of cytokines and their receptors, immune checkpoint molecules, immune co-stimulators, and MHC-related antigen-presenting molecules in all-integrated tumor samples. (Wilcoxon test ***P < 0.001) (**D**) Boxplot of the score of cuproptosis in patients with different tumor cell proportions (TC), with TC0 being the lowest (0–1%), TC2 + being the highest ( > = 50%) and TC1 being in between 1% − 49%. **E** The correlation values between cuproptosis scores and immune-functional signature scores in pan-cancer. **F** The heatmap of the score of cancer-immunity cycle-related pathways in patients with cuproptosis-high and -low groups. The color shows the GSVA score of each cancer-immunity cycle-related pathways in every patient

The immune function mediated by multiple components in TME directly governs antitumor immunity, such as MHC molecule-mediated antigen presentation and interferon (IFN)-mediated cancer cell killing. To investigate why patients with low cuproptosis score exhibited favorable outcomes following immunotherapy, we performed multi-dimension analysis of the cohort from TCGA to compare various immune functions between the two groups. Cytokines and their receptors are essential components of TME, with chemokines responsible for the migration of immune cells into TME, interleukins (ILs) enriched in the TME as immunomodulatory cytokines, and IFNs mediating antitumor activity of effector T cells [20, 21]. Our results showed that gene ontology (GO) terms regarding chemokines, ILs, IFNs, other cytokines, and their receptors were enriched in cuproptosis-low group in most cancer types, with STAD showing distinct patterns (Fig. 2A). This cancer type-specific variation suggests that cuproptosis-immunity relationships may differ across tumor contexts. Similarly, GO terms regarding MHC molecule, immune receptor, antigen stimulus or processing, integrin, immunoglobulin, and molecular mediator of immune response were enriched in cuproptosis-low group (Fig. 2A), which function as crucial immune modulators in TME, suggesting that patients with low cuproptosis scores exhibit superior immune functions. Given the limited sample size of SKCM and the absence of SKCM samples receiving immunotherapy in TCGA, we selected some GEO data for analysis and validation. As shown in Fig. 2A and S1A, most those GO terms were enriched in SKCM samples with low cuproptosis score, an underpinning for the better immunotherapy outcomes in cuproptosis-low group. Consistently, KEGG enrichment analysis showed that chemokine and IL-17 signaling pathways, receptor-related signaling pathways, antigen processing and presentation, cell adhesion molecules, and PD−L1 expression and PD − 1 checkpoint pathway in cancer were activated in the cuproptosis-low group in all-integrated tumor samples (Fig. S1B). Interestingly, diseases associated with hyperimmune were also activated in the cuproptosis-low group (Fig. S1C), suggesting the hyperactive immune function in patients with low cuproptosis scores. Likewise, Enrichment of hallmark pathways showed that IFN-related responses, inflammatory response, and allograft rejection were enriched in the cuproptosis-low group in the majority of cancer types analyzed, while STAD demonstrated different enrichment patterns (Fig. S1D). To validate such enrichment in cuproptosis-low group, we performed differential analysis to identify differential transcriptional features in the high- and low-cuproptosis groups (Fig. 2B). Consistently, cuproptosis score are negatively correlated with the expression of cytokines and their receptors, immune checkpoint molecules, immune co-stimulators, and MHC-related antigen-presenting molecules in most cancer types (Fig. 2B). Likewise, in all-integrated tumor samples, low-cuproptosis group demonstrated higher fraction of those immune mediators or molecules than high-cuproptosis group (Fig. 2C), favoring improved antigen presentation, immune cell chemotaxis and activation, and cancer cell recognition and elimination in patients with low cuproptosis scores.

Considering the importance of PD-1, PD-L1, and CTLA4 in immunotherapy, we visualized the expression of these checkpoints between the two groups in a wide range of cancers, and consistently, the expression of these checkpoints was higher in low-cuproptosis group in most cancer types (Fig. S1E), underlying the improved benefit from immune CPIs. We then analyzed PD-1/PD-L1 expression in IMvigor dataset to verify the difference between two groups. The cuproptosis score was negatively correlated with PD-L1 expression in tumor cells and PD-1 expression in immune cells (Fig. 2D). Furthermore, the immune-functional signature scores were adversely correlated with cuproptosis score in most cancers, consistent with Enrichment of immune function-related pathways and productions in patients with low cuproptosis scores (Fig. 2E). Notably, while patients in the low-cuproptosis group exhibited robust immune infiltration and activation of immune-related pathways, we also observed significantly higher expression of immune checkpoint molecules, including PD-1, PD-L1, and CTLA4, in this group across most cancer types (Fig. S1E). This constellation of features—high immune infiltration coupled with elevated checkpoint expression—may initially appear paradoxical. However, it is consistent with a phenotype of T-cell exhaustion within an inflamed tumor microenvironment [22]. Chronic antigen stimulation in tumors with active immune responses often leads to sustained upregulation of inhibitory receptors on effector T cells, marking them as exhausted but potentially reversible by immune checkpoint blockade [23]. Therefore, the elevated checkpoint expression in the low-cuproptosis group likely reflects a pre-existing but functionally restrained adaptive immune response. This ‘inflamed/exhausted’ phenotype provides a strong rationale for the enhanced clinical benefit observed in these patients following immunotherapy, as these pre-existing tumor-infiltrating lymphocytes are poised to be reinvigorated by anti-PD-1/PD-L1 therapy. In contrast, the high-cuproptosis group, characterized by immune desert phenotypes and lower checkpoint expression, may lack the target T-cell population necessary for such therapeutic response. Consistently, we observed significantly higher cytotoxicity and exhaustion scores in the cuproptosis-low group compared to the cuproptosis-high group (Fig. S1F).

Subsequently, we assessed the cancer-immunity cycle-related pathways in both groups. The antitumor immunity generally involves a series of stepwise events, known as cancer-immunity cycle, to eliminate cancer cells [24]. As shown in Fig. 2F, a majority of steps were more active in low-cuproptosis group than in high-cuproptosis group, further supporting the potent antitumor immune system in patients with low cuproptosis scores. Overall, cuproptosis seems to be a barrier to immune function and antitumor immunity, whereas low-cuproptosis TME is characterized by an abundance of immune-boosting molecules and mediators, which is an underlying reason for patients with low cuproptosis to benefit tremendously from immunotherapy.

### Extrinsic immune landscapes: immune infiltration in cuproptosis-low and -high groups

**Fig. 3:**
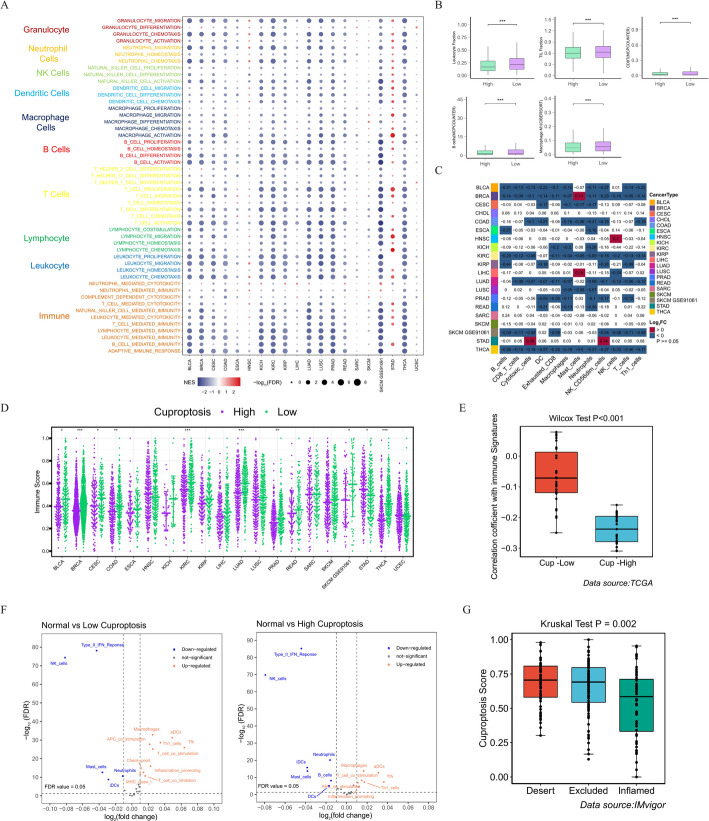
Immune cell infiltration in cuproptosis-low and -high groups. **A** Enrichment of GO pathways associated with immune infiltration in patients with cuproptosis-high and -low groups from TCGA datasets. **B** Boxplot of the fraction of immune-associated features including Leukocytes, TIL, CD8T cells, B cells and macrophage M1 cells in patients with cuproptosis-high and -low groups. (Wilcoxon test ***P < 0.001) (**C**) The fold differences of immune infiltration scores from Danaher et al. in patients from cuproptosis-high and -low groups. **D** Jitter plot of the immune score calculated by ‘ESTIMATE’ R package in patients with cuproptosis-high and -low groups. Horizontal bars show median score value. (Wilcoxon test *P < 0.05; **P < 0.01; ***P < 0.001). **E** Boxplot of the correlation coefficients of cuproptosis score and immune infiltration-function scores in cuproptosis-high and -low groups. **F** Volcano plots of differentially expressed immune signatures between normal and different cuproptosis groups. **G** Boxplot of the score of cuproptosis in patients with different inflamed-immunophenotypes

The pronounced distinctions in immune function between cuproptosis-low and -high groups motivated us to investigate the landscapes of immune cell infiltration between the two groups. A variety of immune cells contribute substantially to TME and antitumor immunity, and therefore we initially explored immune infiltration at the genomic level in two groups of patients. In keeping our suspicion that cuproptosis may dampen immune cells, GO terms showed that diverse immune cell-associated development, migration, activation, and immunity are enriched in cuproptosis-low group in all cancer types except STAD (Fig. 3A). Consistently, most those GO terms were enriched in SKCM samples with low cuproptosis score from GEO database (Fig. S2A). Similarly, there was a greater abundance of leukocytes, tumor-infiltrating lymphocytes (TILs), CD8 T lymphocytes, B cells, or M1 macrophage in the low-cuproptosis group compared to the high-cuproptosis group in all-integrated tumor samples (Fig. 3B). Specific to immune infiltration by cancer type, those immune cells were more enriched in low-cuproptosis group than those in high-cuproptosis group in many cancer types, such as BLCA, BRCA, KIRC, or SKCM (Figs. S2B–F). Interestingly, immunosuppressive cells, such as M2 macrophages and MDSCs, positively correlated with cuproptosis in many cancers, further supporting the remarkable immune function and antitumor immunity in patients with low cuproptosis scores (Figs. S2G, H). To fully understand the correlation of cuproptosis with immune-infiltrating profile, we compared the distribution of various immune cells between two groups based on the immune infiltration scores from Danaher et al. Consistently, a wide range of immune cells, such as antigen-bearing cells, innate immune cells, B cells, and T cells, are negatively associated with cuproptosis scores in most cancers (Fig. 3C), suggesting that cuproptosis is possibly detrimental to the infiltration of immune cells in TME.

To validate the immune infiltration, we used ESTIMATE algorithm in high- and low-cuproptosis group, which reflects the infiltration of immune cells. Consistently, in most cancer types, patients with low cuproptosis scores exhibited increased ESTIMATE score and immune score compared to patients with high cuproptosis scores (Fig. 3D). To delve deeper into the link between cuproptosis score and both immune infiltration and immune function, we compared the correlation coefficients for cuproptosis and immune signatures between two groups by combined immune infiltration-function analysis. As shown in Fig. 3E, there was a robust negative correlation in high-cuproptosis group (correlation coefficients ranged between approximately − 0.2 and − 0.3.), but not in low-cuproptosis group, suggesting that the higher the cuproptosis score, the stronger the negative correlation between it and immune infiltration-function. Furthermore, the up-regulated immune signatures were dramatically increased in tumor samples from patients with low cuproptosis scores compared to normal tissues (Fig. 3F). This differential correlation pattern suggests a threshold-dependent relationship where high cuproptosis scores create an increasingly immunosuppressive microenvironment that progressively impairs immune infiltration and function, while low cuproptosis scores allow for maintained immune activity with minimal interference from cuproptosis-related pathways.

An innovative insight is to link the patient’s immune phenotype, including inflamed, immune-excluded, and immune-desert, to cuproptosis score [7]. The inflamed-immunophenotype generally respond well to immunotherapy, while exclude or desert-immunophenotype are relevant to poor efficacy [7]. Interestingly, patients with inflamed-immunophenotypes presented significantly lower cuproptosis scores than those with desert- or excluded-immunophenotypes, and the immune-desert patients displayed highest cuproptosis scores (Fig. 3G). Given that the inflamed-immunophenotype are featured with IFNγ signaling, robust immune infiltration, and high PD-L1 expression, whereas immune cells are deficient in desert-immunophenotype [7], these results are consistent with the correlation of cuproptosis with TME and immuno-response. Collectively, our results depict an extrinsic immune landscape associated with cuproptosis in pan-cancer, suggest cuproptosis as a modulator of antitumor immunity by affecting a variety of components in the TME, and provide a robust explanation for the optimal immunotherapy outcomes in patients with low cuproptosis scores.

### Cuproptosis-related immune landscapes at single-cell and two-dimensional spatial level

**Fig. 4:**
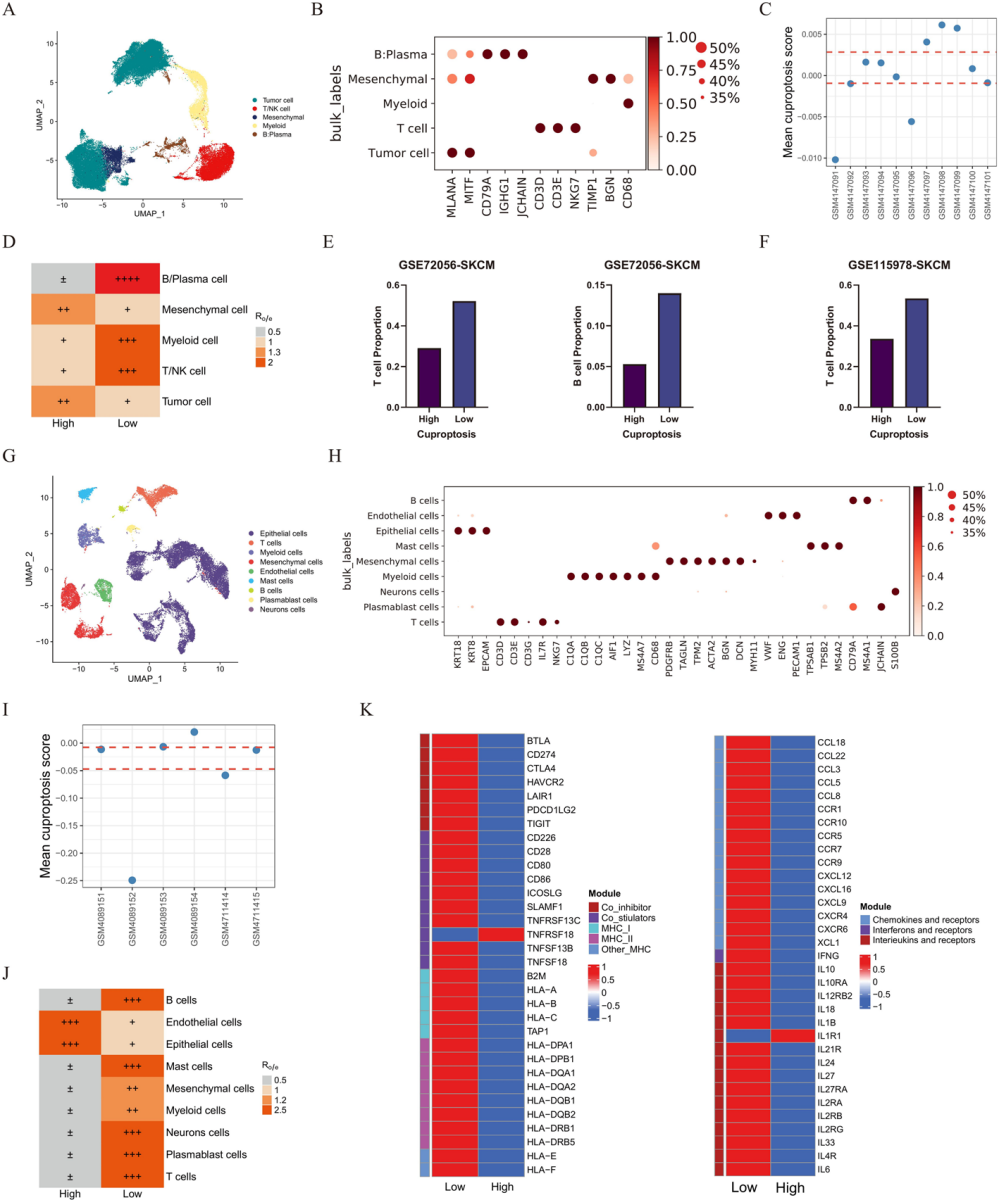
Cuproptosis-related extrinsic immune landscapes at single-cell resolution. **A** UMAP plot showing the composition of 5 main subtypes derived from uveal melanoma tumor tissues (GSE139829). **B** The expression of markers for 5 cell types. The color of each dots represents the mean expression of marker gene within each cell type and the size of each dots shows the fraction of cells expressing the marker gene in this cell type. **C** Dot plot showing the mean cuproptosis score for each patient sample in the melanoma tumor tissues (GSE139829). **D** Heatmap depicting the sample preference of each cell type in GSE139829 dataset, quantified by the Ro/e score. **E**, **F** The proportion of each immune cell type in multiple single-cell datasets. **G** UMAP plot showing the composition of 5 main subtypes derived from BLCA single-cell RNA-seq dataset (GSE7205663). **H** The expression of markers for 9 cell types. The color of each dots represents the mean expression of marker gene within each cell type and the size of each dots shows the fraction of cells expressing the marker gene in this cell type. **I** Dot plot showing the mean cuproptosis score for each BLCA samples from GSE7205663 dataset. **J** Heatmap depicting the sample preference of each cell type in GSE7205663 dataset, quantified by the Ro/e score. **K** The mean expression of cytokines, MHC molecules, immune checkpoints, or immune co-stimulators at single-cell resolution in cuproptosis-high and -low samples from GSE137829 dataset. The mean expression of each gene were normalized to a range of -1 to 1 in each row

Considering the advantages of single-cell profiling technology in better characterizing intra-tumor heterogeneity, we further investigated correlations between cuproptosis and immune infiltration-function at single-cell resolution. Employing graph-based principal component clustering combined with marker-based annotations, we annotated 5 major cell types for eleven uveal melanoma tumor tissues from the GSE139829 dataset using combinations of biomarkers: Melan-A (MLANA) and Melanocyte Inducing Transcription Factor (MITF) (tumor cells); CD3 Delta Subunit Of T-Cell Receptor Complex (CD3D), CD3 Epsilon Subunit Of T-Cell Receptor Complex (CD3E) and Natural Killer Cell Granule Protein 7 (NKG7) (T/NK cells); TIMP Metallopeptidase Inhibitor 1 (TIMP1) and Biglycan (BGN) (mesenchymal cells), CD68 (myeloid cells); CD79A, Immunoglobulin Heavy Constant Gamma 1 (IGHG1) and Joining Chain Of Multimeric IgA And IgM (JCHAIN) (B: Plasmablast cells). We calculated the mean cuproptosis score for each patient and stratified samples into cuproptosis-low and cuproptosis-high groups based on quartile distribution, where the first quartile (Q1) represented the cuproptosis-low group and the fourth quartile (Q4) represented the cuproptosis-high group. (Figs. 4A–C). We found that T/NK cell proportion or myeloid cell proportion was dramatically decreased in cuproptosis-high group (Fig. 4D). Similarly, T cell proportion, NK cell proportion, or B cell proportion was higher in cuproptosis-low group than in cuproptosis-high group, in terms of GSE72056 or GSE115978 (Figs. 4E, F). We also found consistent results in BLCA single-cell RNA-seq dataset (GSE7205663; Figs. 4G–J). T cell proportion, NK cell proportion, or B cell proportion was dramatically increased in cuproptosis-low group (Figs. 4G–J). To assess the expression of cytokines, MHC molecules, immune checkpoints, or immune co-stimulators at single-cell resolution, we performed differential analysis and selected significant different transcriptional features. Consistently, expression of these TME components was enriched in cells with low cuproptosis scores (Fig. 4K). Collectively, correlations between cuproptosis scores and extrinsic immune landscapes by single-cell analysis are consistent with those in TCGA or other databases, and importantly, the predominant enrichment of immune cells and molecules in cuproptosis-low group at single-cell resolution microscopically decodes the advantages of immunotherapy in patients with low cuproptosis scores.

**Fig. 5:**
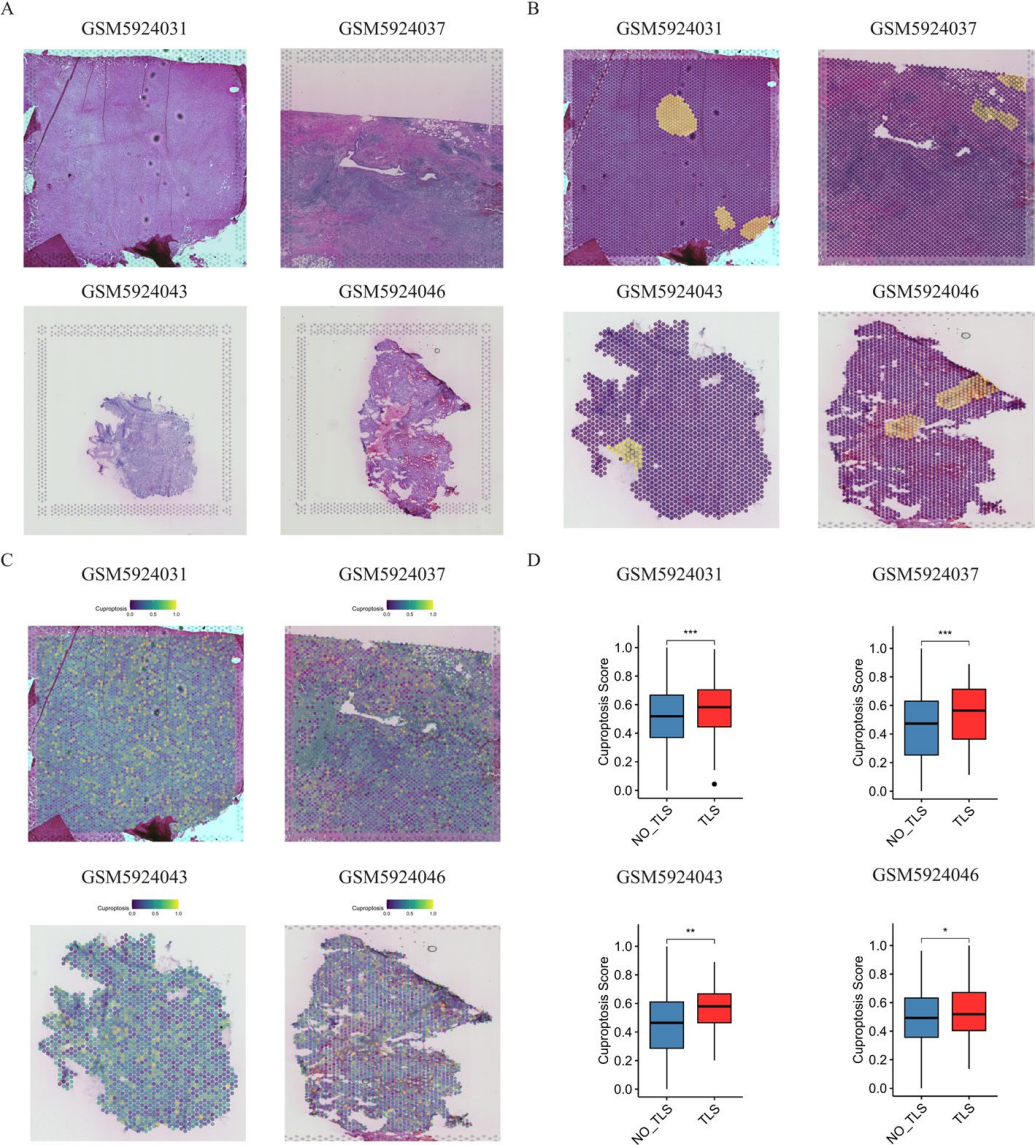
Immune landscapes associated with cuproptosis at spatial transcriptome resolution. **A** Hematoxylin and eosin (H&E) plot of each sample. **B** Spatial labeling of tumor-infiltrating lymphocytes (TILs) and tumor regions. The spots labeled in yellow represent TIL regions, while the remaining spots correspond to tumors. **C** Cuproptosis scores for each spot. **D** Boxplot showing the distribution of cuproptosis scores within TIL and tumor regions

**Fig. 6:**
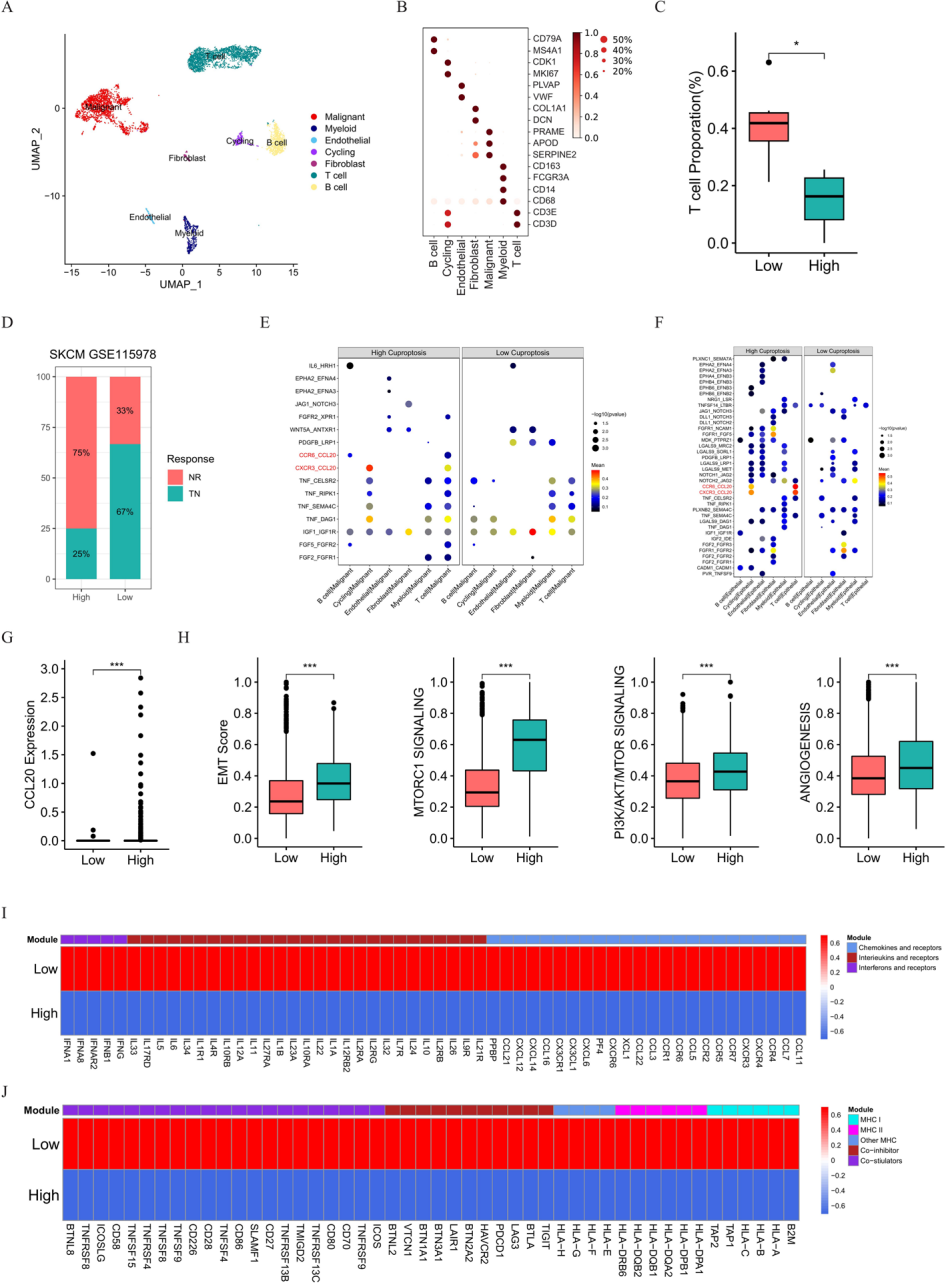
Cuproptosis-related immune landscapes at single-cell resolution upon immunotherapy. **A** UMAP plot showing the composition of 7 main subtypes derived from melanoma tumor tissues (GSE115978). **B** The expression of markers for 7 cell types. The color of each dots represents the mean expression of marker gene within each cell type and the size of each dots shows the fraction of cells expressing the marker gene in this cell type. **C** Boxplot of the proportion of T cells in cuproptosis-high and -low groups. (Wilcoxon test *P < 0.05) (**D**) The proportion of response status (TN: Treatment naive, NR: non-response) to immunotherapy in cuproptosis-high and -low groups. **E** Cellular communication in BCC patients with cuproptosis-high and -low groups. **F** Cellular communication in SKCM patients with cuproptosis-high and -low groups. **G** Boxplot of the expression of CCL20 in cuproptosis-high and -low groups. (Wilcoxon test ***P < 0.001). **H** Boxplot of the enrichment of EMT, MTORC1, PI3K/AKT/MTOR and ANGIOGENESIS in cuproptosis-high and -low groups. (Wilcoxon test ***P < 0.001). **I**, **J** The mean expression of cytokines, MHC molecules, immune checkpoints, or immune co-stimulators at single-cell resolution in cuproptosis -high and -low samples from GSE115978 dataset. The mean expression of each gene were normalized to a range of -1 to 1 in each row

To examine the immune landscapes related to cuproptosis at a two-dimensional spatial level, we analyzed four spatial transcriptome datasets containing distinct tertiary lymphatic structures (TILs) labels and then calculated the cuproptosis score for each spot (Fig. 5A–C). Consistently, the cuproptosis scores of immune cell regions, specifically tertiary lymphoid structures (TLS), were consistently higher than those of tumor cell regions, as shown in Fig. 5D. This suggests that immune cells may be more susceptible to cuproptosis than tumor cells. These findings offer a possible explanation for the reduced immune cell infiltration and impaired immune function observed in patients with high cuproptosis scores. To further explore cuproptosis-related immune landscapes at single-cell resolution upon immunotherapy, we examined two single-cell datasets from SKCM and BCC patients undergoing immunotherapy treatment. Each cell was labeled based on classical signatures to allow for more precise analysis (Figs. 6A, B; Figs. S3A-B). Consistently, T cell proportion was dramatically decreased in cuproptosis-high group receiving immunotherapy (Fig. 6C; Fig. S3D), and correspondingly, the cuproptosis-high group exhibited increase non-response after immunotherapy (Fig. 6D; Fig. S3C). Further analysis revealed that the activation of CXCR3-CCL20 (or CCR6-CCL20)-mediated cell communication was observed in the SKCM or BCC patients with high cuproptosis scores (Figs. 6E, F). Additionally, there was an increase in CCL20 expression in the SKCM cuproptosis-high group (Fig. 6G). It has been established that CXCR3/CCR6-CCL20 plays an immunosuppressive role in anticancer immunity and promotes cancer progression [25, 26], consistent with the immunosuppressive TME and impaired anticancer immunity in patients with high cuproptosis scores. Correspondingly, downstream factors of CCL20 were increased in cuproptosis-high group, including epithelial-to-mesenchymal transition (EMT), PI3K-AKT-mTOR signaling, and angiogenesis (Fig. 6H), which underlie the CCL20-mediated cancer progression.

In addition, in line with the compromised efficacy of immunotherapy, the levels of chemokines, interleukins, interferons, MHC molecules and checkpoints were all decreased in cuproptosis-high group receiving immunotherapy (Figs. 6I, J).

### Potential intrinsic immune profiles in the patients with high and low cuproptosis scores

**Fig. 7:**
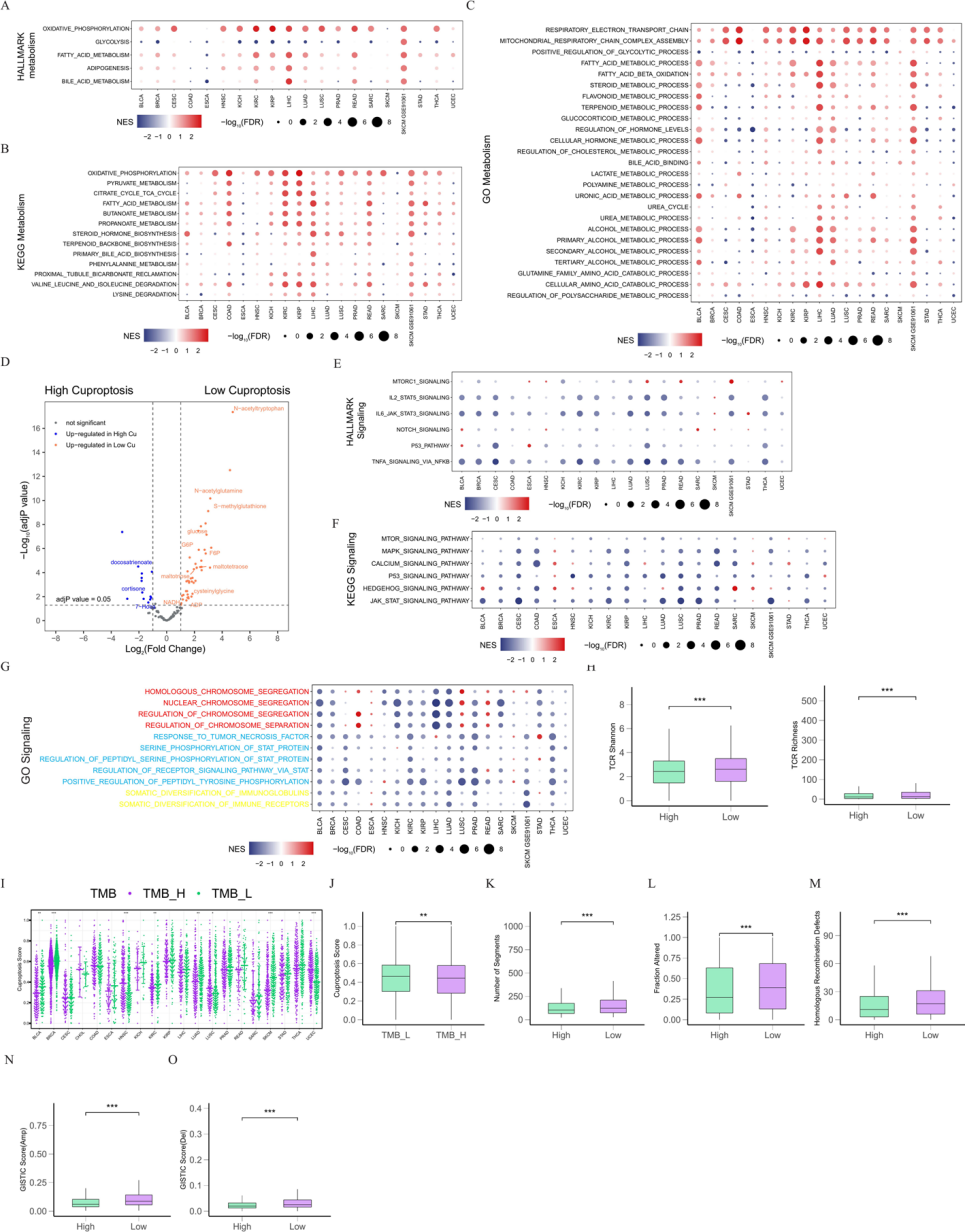
Potential intrinsic immune profiles in patients with high and low cuproptosis scores. **A** Enrichment of hallmark pathways associated with metabolism in patients with cuproptosis-high and -low groups from GEO and TCGA datasets. **B** Enrichment of KEGG pathways associated with metabolism in patients with cuproptosis-high and -low groups. **C** Enrichment of GO pathways associated with metabolism in patients with cuproptosis-high and -low groups. In panels **A**–**C**, if the NES value was less than 0, the pathway was considered enriched in the cuproptosis-low group; otherwise, the pathway was considered enriched in the cuproptosis-high group. **D** Volcano plots of the difference of metabolomics characterize in cuproptosis-high and -low groups. **E**–**G** Enrichment of signaling pathways from HALLMARK, KEGG and GO datasets in patients with cuproptosis-high and -low groups. If the NES value was less than 0, the pathway was considered enriched in the cuproptosis-low group; otherwise, the pathway was considered enriched in the cuproptosis-high group. **H** Boxplot of TCR diversity including TCR shannon and TCR richness in patients with cuproptosis-high and -low groups. (Wilcoxon test ***P < 0.001) (**I**) Jitter plot of cuproptosis score in TMB-low and -high groups patients. (Wilcoxon test *P < 0.05; **P < 0.01; ***P < 0.001) (**J**) Boxplot of cuproptosis score in TMB-low and -high groups all-integrated tumor samples. (*Wilcoxon test *P < 0.01) (K) Boxplot of segment number in cuproptosis-low and -high groups in all-integrated tumor samples. (Wilcoxon test ***P < 0.001) (**L**) Boxplot of the altered fraction in cuproptosis-low and -high groups in all-integrated tumor samples. (Wilcoxon test ***P < 0.001) (**M**) Boxplot of the homologous recombination defects score in cuproptosis-low and -high groups in all-integrated tumor samples. (Wilcoxon test ***P < 0.001) (**N**) Boxplot of the amplification GISTIC score in cuproptosis-low and -high groups in all-integrated tumor samples. (Wilcoxon test ***P < 0.001) (**O**) Boxplot of the deletion GISTIC score in cuproptosis-low and -high groups in all-integrated tumor samples. (Wilcoxon test ***P < 0.001). Data source for panels J-O is all-integrated tumor samples from TCGA

We subsequently investigate potential reasons for advantageous extrinsic immune landscapes in patients with low cuproptosis scores. Given the cuproptosis as a metabolism-modulated cell death, we first analyzed the metabolite profiles or metabolic processes between two groups from HALLMARK, KEGG or GO datasets which were downloaded from Msigdb database. We found that the ETC, OXPHOS, pyruvate metabolism, and TCA cycle are active in cuproptosis-high group (Figs. 7A–C, S4A), in line with the metabolic features of cuproptosis [12]. Interestingly, fatty acid metabolism and fatty acid oxidation (FAO) are activated in cuproptosis-high group in most cancers (Fig. 7C) and SKCM tumor cells (Fig. S4B). It has been well-established that OXPHOS or FAO is the predominant energy source in immunosuppressive cells, such as immunosuppressive macrophage or Treg cells [8, 16]. Therefore, the upregulated OXPHOS or FAO was associated with the poor response to immunotherapy in patients with high cuproptosis scores. Consistent with the activated fatty acid metabolism, the adipogenesis and short-chain fatty acids, such as butanoate and propionate, were increased in cuproptosis-high group (Figs. 7A, B), contributing the immunodeficient TME and immune-desert phenotype [7].

Steroids (e.g., steroid hormone, flavonoids, terpenoids, and glucocorticoids) and cholesterol have a major role in immunosuppression and anti-inflammation [7, 27, 28], which were positively correlated with cuproptosis in most cancers (Figs. 7B, C). Similarly, other immunosuppressive metabolites or metabolic processes were elevated in cuproptosis-high group, including bile acids, lactate, polyamines, phenylalanine, urea cycle, uronic acid, and alcohol (Figs. 7A–C). The bicarbonate reclamation (Fig. 7B), together with the accumulation of those acids, promoted an acidic TME that fosters immune evasion in patients with high cuproptosis scores [7]. Moreover, since multiple amino acids are essential for the proliferation and function of immune cells [8, 16], the down-regulated catabolic process of glutamine family amino acid and cellular amino acid, as well as the attenuated degradation of valine, leucine, isoleucine, and lysine, promoted the normal supply of these amino acids to immune cells in cuproptosis-low group (Figs. 7B, C). Besides, the immune-boosting polysaccharide was enriched in cuproptosis-low group (Fig. 7C). Notably, we found consistent enrichment results in SKCM samples including GSE133713, GSE157738, GSE22153S and GSE98394 from GEO database (Figs. S5A–C).

To directly examine the metabolite enrichment between two groups, we analyzed an HCC cohort (GSE76297) with transcriptomics and metabolomics measured simultaneously [29]. Transcriptomics was used to recognize cuproptosis-low and -high groups. Similarly, metabolomics showed that glycolytic process (e.g., glucose, glucose-6-phosphate (G6P), fructose-6-phosphate (F6P)), maltose (can be converted to glucose), and various amino acids are upregulated in cuproptosis-low group, whereas immunosuppressive steroids (e.g., cortisone, 4-androsten-3beta,17beta-diol disulfate), 7-Hoca (cholesterol), or docosatrienoate (fatty acid) are enriched in cuproptosis-high group (Fig. 7D), in line with cuproptosis-related metabolic patterns and immune response. Consistently, the accumulation of Adenosine-diphosphate (ADP) and Nicotinamide adenine dinucleotide (NADH) in cuproptosis-low group potentially suggests a relatively inactive ETC and OXPHOS (Fig. 7D). Collectively, the predominant energy supply pattern by oxidative phosphorylation and FAO, coupled with the leading immunosuppressive metabolic processes and the absence of metabolites favoring immunity, is possibly detrimental to the proliferation and function of effector T cells, B cells, and antigen-promoting cells. This metabolic configuration likely contributes to the dampened immune landscape in patients with high cuproptosis scores. Conversely, more pro-immunity substances, fewer immunosuppressive, and stronger glycolysis metabolites may underlie the superior immune landscape in patients with low cuproptosis scores.

Cuproptosis-associated metabolic characteristics can also affect several signaling pathways. We observed a pan-cancer negative correlation between cuproptosis score and NOTCH signaling or mTOR signaling (Figs. 7E, F), which is reported as a consequence of amino acid deficiency or glycolytic restriction [30]. In addition, immune response-regulating signaling pathway, p53 signaling, MARK signaling, Rap1 signaling, TNF signaling, NF-kappa B signaling, JAK-STAT3 signaling, STAT5 signaling, hedgehog signaling pathway, and calcium signaling were activated in pan-cancer patients with low cuproptosis scores, contributing to the immune-boosting TME (Figs. 7E, F). Likewise, the phosphorylation of serine or tyrosine is important for immunosignal transduction, which were activated in cuproptosis-low group (Fig. 7G). Moreover, patients with low cuproptosis scores seem to have enriched TCR diversity or somatic diversification of immunoglobulins or immune receptors (Figs. 7G, H), facilitating recognition and killing of cancer cells. Intriguingly, patients with low cuproptosis scores seem to have increased TMB (especially BLCA, BRCA, KIRC, and SKCM), Copy Number Variation (CNV) burden, homologous recombination defects, focal or broad copy number aberrations, and chromosomal aberrations (Figs. 7I–O, S5D–H). Overall, activated immune-related signaling pathways and genetic characteristics favoring antitumor immunity likewise reveal potential mechanisms for advantageous extrinsic immune landscape in patients with low cuproptosis scores.

## Discussion

Cuproptosis is a copper-induced RCD triggered by a toxic gain of function in mitochondria owing to the binding of copper to lipoylated TCA cycle proteins [12]. Previous studies suggest a paradoxical function of copper in antitumor immunity [13–15]. In addition, cuproptosis-related TCA cycle, OXPHOS or fatty acid metabolism is inextricably linked to TME and antitumor immunity [7, 8, 16]. Therefore, a comprehensive multi-omics analysis of the differences in cuproptosis between normal and cancerous tissues and the relevance of cuproptosis to immune landscape and immunotherapeutic benefit will substantially contribute to the understanding of cuproptosis in terms of onco-immunology, identifying those populations with potentially optimal efficacy, as well as developing underlying therapeutic strategies. Considering that cuproptosis was proposed this year and the lack of systematic pan-cancer analysis, our study established a robust cuproptosis scoring model based on genes directly governing cuproptosis and explored its clinical links to anticancer immunity in a multi-omics context across a wide range of cancers.

RCD has been linked to the release of cancer antigens, interferon (IFN) activity, or immune cell damage [11]. However, previous studies reported a dual role of copper in antitumor immunity, and cuproptosis-related mitochondrial metabolism also contributes to the regulation of TME [7, 8, 13–15]. Intriguingly, our pan-cancer analysis revealed that patients with low cuproptosis scores exhibited superior immunotherapy outcomes. Notably, we suggested cuproptosis score as an independent predictor for immunotherapy benefit, even more pronounced than other canonical immunotherapy biomarkers, including TMB, immune checkpoints, and immune phenotype. Incorporating TMB can further improve the stratification of cancer patients by cuproptosis score, suggesting that the combination of cuproptosis and other biomarkers has promising clinical applications for immunotherapy. Therefore, our results seem to be consistent with the previous reports that copper depletion promotes immunotherapy and active mitochondrial metabolism contributes to immune evasion.

Above-mentioned results raised an interesting question as to why cuproptosis negatively correlates with immunotherapy outcomes. We speculate that cuproptosis could kill immune cells and cancer cells simultaneously, similar to other types of RCD. For instance, immune cells are extremely vulnerable to ferroptosis, and ferroptosis inducers administered in vivo can in parallel kill immune cells and tumor cells [17, 18]. To demonstrate our speculation, we performed single-cell omics and transcriptomic analysis using TCGA and GEO database. Pan-cancer multi-omics analyses revealed that immune cell functions were diminished in cuproptosis-high group, such as decreased MHC-mediated antigen processing, production of cytokines or immunoglobulins, expression of immune checkpoints, or immune receptor signaling. We further showed that immune cell infiltration was significantly decreased in the cuproptosis-high group, while immunosuppressive cells were increased in cuproptosis-high group in many cancer types. Cuproptosis-high group exhibited opposite results, consistent with the previous report that copper depletion increased tumor-infiltrating T cells and NK cells and decreased immunosuppressive cells [14, 15]. Besides, there is a possibility that cancer cells dying from cuproptosis impede antigen-presenting cell-mediated anti-tumor immunity, as was observed in ferroptosis (another metabolism-mediated RCD) [19]. Consequently, dampened immune infiltration and function offers a rational explanation for poor immunotherapy benefit in patients with high cuproptosis scores.

The following question is why cuproptosis negatively correlates with immune infiltration and function. On the one hand, spatial transcriptomics reveals that immune cells exhibits higher cuproptosis scores than tumor cells, indicating that immune cells are more vulnerable to cuproptosis than tumor cells; on the other hand, glycolysis and mitochondrial metabolism play an important role in the survival and function of immune cells [8, 16]. Effector T cells, B cells, DC cells, M1 macrophages, and NK cells are more dependent on glycolysis for energy supply, whereas immunosuppressive M2 macrophages and MDSCs are mainly supported by OXPHOS or FAO in mitochondria [8, 16]. Since cuproptosis is governed by mitochondrial metabolism, cuproptosis-related metabolic features may be preferable for the growth and function of immunosuppressive cells. Our transcriptomic and metabolomic analysis uncovered that cuproptosis-high group was linked to active OXPHOS and fatty acid metabolism and decreased glycolysis, consistent with the cuproptosis-related metabolic profiles [12]. Remarkably, antitumor-promoting immune substances, such as diverse amino acids and polysaccharides, were enriched in cuproptosis-high group, while immunosuppressive chemicals, such as steroid hormones and acidic substances, were abundant in cuproptosis-low group [7, 8, 16], further explaining the gap in extrinsic immune landscape and immunotherapy outcomes between the two groups. Furthermore, antitumor-promoting signaling, TCR diversity, TMB, CNVs, or chromosomal aberrations were decreased in cuproptosis-high group, which may be relevant to cuproptosis-related metabolic profiles, as another potential reason for inferior TME and immune responses in patients with high cuproptosis scores.

## Limitations

This study has several limitations. First, the limited availability of publicly accessible immunotherapy transcriptomic data necessitates further validation of the association between cuproptosis and immunotherapy prognosis in larger population cohorts. Additionally, our study’s metabolomics data focused solely on individual cancer types, which may not accurately represent the pan-cancer metabolic characteristics associated with cuproptosis. Furthermore, the current scarcity of immunotherapy single-cell datasets containing tumor cells calls for further investigation into the relationship between cuproptosis and the tumor microenvironment in immunotherapy patients, specifically through ligand-receptor interaction analysis to provide additional predictive insights. Notably, our study reveals important cancer type-specific variations in cuproptosis-immunity relationships. While the majority of cancer types demonstrated consistent patterns supporting our main conclusions, STAD showed distinct characteristics that differed from other tumor types. This heterogeneity likely reflects the diverse metabolic landscapes and immune microenvironments across different cancer contexts. Future studies should investigate the underlying mechanisms driving these cancer type-specific differences, particularly focusing on tissue-specific copper metabolism and immune cell vulnerabilities.

## Conclusion

In conclusion, our multi-omics pan-cancer analysis characterizes cuproptosis score in a wide range of cancers and we established cuproptosis score as an independent predictor for immunotherapy outcome. Further transcriptomics, single-cell omics, spatial transcriptomics, and metabolomics depict the extrinsic and intrinsic immune landscapes, provide important insights into the gap in immunotherapy outcomes between the two groups. To the best of our knowledge, this is the first study to comprehensively analyze the cuproptosis score in pan-cancer, providing a broad framework for understanding the relevance of cuproptosis to immuno-oncology, clinical benefits and applications.

## Methods

### Pan-cancer datasets collection

Transcriptome datasets combined with clinical data (e.g., age, gender, stage, overall survival, progression-free survival) from 23 cancers were downloaded from the TCGA data portal (https://portal.gdc.cancer.gov/). The richness of T cell receptor/B cell receptor (TCR/BCR) among 23 cancer samples were available at https://gdc.cancer.gov/about-data/publications/panimmune. GEO dataset (SKCM: GSE91061), containing normal and tumor samples was obtained from Gene-Expression Omnibus (GEO) (https://www.ncbi.nlm.nih.gov/geo/) for further analysis. In addition, other 4 SKCM GEO datasets including GSE133713 [31], GSE157738 [32], GSE22153 [33] and GSE98394 [34] were obtained from GEO to verify the immune characteristics of SKCM. All datasts were shown in Table S1. The data was analyzed using R (version 4.1.1) and R Bioconductor packages.

### Immunotherapy-associated datasets collection

Multiple datasets with anti-PD-L1/PD-1/CTLA4 cohort were collected in the study to investigate the association between immunotherapy efficacy, prognosis and cuproptosis score. The Lauss cohort [35] (GSE100797: adoptive T cell therapy treated melanoma), the Ascierto cohort [36] (GSE79691: Anti-PD-1 treated melanoma), the Ascierto cohort [37] (GSE67501: Anti-PD-1 treated renal cell carcinoma), the Birkbak cohort [38] (GSE103668: neoadjuvant platinum & bevacizumab treated triple negative breast cancer) and the Kim cohort [39] (GSE19423: intravesical therapy treated primary bladder cancer) were obtained from GEO. The Liu/VanAllen cohort [40, 41] (phs000452.v3: anti-PD1/CTLA4-treated metastatic melanoma) was downloaded from dbGaP database (https://www.ncbi.nlm.nih.gov/gap/). The Necchi cohort [42] (IMvigor210: Atezolizumab treated advanced or metastatic urothelial carcinoma) was downloaded using “IMvigor210CoreBiologies” R package. Gene expression and clinical information of these datasets with immunotherapy cohort were collected.

### Single cell datasets collection

Processed gene expression profiles for uveal melanoma and prostate cancer were retrieved from GEO under accession numbers GSE72056 [43] (4645 single cells profiles including malignant, B, Cancer-Associated Fibroblasts (CAFs), endothelial, T and NK cells from melanoma tumor), and GSE115978 [44] (7186 immune cells from 31 tumor samples of melanoma patients treated with checkpoint inhibitors), and GSE137829 [45] (26179 single cells profiles from 6 neuroendocrine prostate cancer). Additionally, for investigate the mechanism of copper death affecting immunotherapy effect, we obtained one single cell datasets from patients undergoing immunotherapy under accession numbers GSE139829 [46] (59,915 single cells isolated from 8 primary and 3 metastatic melanoma patients) and GSE123814 [47] (51775 single cells isolated from 11 basal cell carinoma patients).

### Spatial dataset collection

To investigate the correlation between cuproptosis and immune microenvironment, we analyzed four spatial transcriptome datasets that had clear labels of tertiary lymphatic structures (TILs). These datasets can be accessed from GEO under accession numbers GSE72056.

### The cuproptosis model construction and cuproptosis status evaluation

The cuproptosis model was established based on the expression data for genes of core cuproptosis-promoting components (pro-CUPs), including FDX1, LIAS, LIPT1, DLD, DLAT, PDHA1 and PDHB; and anti-cuproptosis-promoting (anti-CUPs) genes, including MTF1, GLS CDKN2A [12]. Applying single sample gene set enrichment analysis (ssGSEA), we calculated enrichment score (ES) of pro-CUPs and anti-CUPs. The cuproptosis score was then calculated by the differences of ssGSEA score between the ES of pro-CUPs and anti-CUPs, as following:

$${\text{Cuproptosis score}}\,=\,{\text{ssGSEA Score }}\left( {{\mathrm{pro}} - {\mathrm{CUPs}}} \right){\text{ }} - {\text{ ssGSEA Score }}\left( {{\mathrm{anti}} - {\mathrm{CUPs}}} \right)$$

### Gene set variation analysis (GSVA) and pathways enrichment analysis

In order to study the differences of different cuproptosis status in cancer hallmark pathways, we used “GSVA” R package [48] to conduct GSVA enrichment analysis. The gene set ‘h.all.v7.2.symbols’, ‘c5.go.v7.5.1.symbols’ and ‘c2.cp.kegg.v7.5.1.symbols’ were retrieved from the MSigDB database (http://software.broadinstitute.org/gsea/msigdb/index.jsp). Pathway enrichment analysis was performed by using the fgsea package (An algorithm for fast preranked gene set enrichment analysis using cumulative statistic calculation) and the clusterProfiler package [49].

### Immune cell infiltration abundance calculation

The infiltration estimation matrices of immune cells of patients with 23 cancers were calculated by CIBERSORT [50], XCELL [51], TIMER [52] and MCPCOUNTER [53] algorithms and all four types of infiltration estimation results were downloaded from Tumor Immune Estimation Resource (TIMER2.0) database (http://cistrome.org/TIMER/download.html). In addition, the proportion of myeloid-derived suppressor cells (MDSCs) was calculated by Tumor Immune Dysfunction and Exclusion (TIDE) website (http://tide.dfci.harvard.edu/) [54].

### The immune signature calculation

We acquired 26 classical immune signatures from the study by He et al., including Antigen-presenting cell (APC) co inhibition, APC co stimulation, B cells, Cytokine-cytokine receptor (CCR), Check-point, Cytolytic activity, Dendritic Cells (DCs), human leukocyte antigen (HLA), Immature Dendritic Cells ) iDCs, Inflammation-promoting, Macrophages, Mast cells, MHC class I, Neutrophils, NK cells, Parainflammation, Plasmacytoid dendritic cells (pDCs), T cell co-inhibition, T cell co-stimulation, Follicular helper T cell (Tfh), Th1 cells, Th2 cells, tumor-infiltrating lymphocyte (TIL), regulatory T cells (Treg), Type I IFN Reponse, Type II IFN Reponse [55]. In addition, 12 immune infiltration signatures were obtained from a previous TCGA pan-cancer study conducted by Danaher et al. [56], which included B cells, T cells, DCs, macrophages, exhausted CD8 T cells, CD8 T cells, neutrophils, cytotoxic cells, NK CD56dim cells, mast cells, NK cells and Th1 cells. Besides, we also collected another 17 immune function genesets including MHCI, MHCII, Co_activation_molecules, Antitumor_cytokines, Checkpoint_molecules, Protumor_cytokines, APC_co_inhibition, APC_co_stimulation, Cytolytic_activity, Inflammation_promoting, MHC_class_I, Parainflammation, T_cell_co_inhibition, T_cell_co_stimulation, Type_I_IFN_Reponse, Type_II_IFN_Reponse, CCR. All these 55 immune-associated genesets were shown in Table S2-S4. In addition, we also downloaded the fraction numbers of leukocytes and tumor-infiltrating lymphocytes (TILs) which were calculated based on H&E images from 13 TCGA tumor types from Saltz et al. [57]. ESTIMATE algorithm [58] was utilized to calculate the ESTIMATE score, which revealed tumor purity, and the immune score of each patients.

### Single cell data processing and cell type annotation

Seurat v.3.0.0 [59] was utilized for data normalization, dimensionality reduction and clustering using default parameters. At the preprocessing stage for melanoma (GSE139829 and GSE123814) and prostate cancer (GSE137829) datasets, cells were filtered based on the criteria of expressing a minimum of 200 genes. Cells with more than 20% mitochondrial gene expression contribution were also removed. We downloaded the cell type annotation information for future analysis for melanoma datasets GSE72056 and GSE115978. Cell clusters were annotated using SingleR [60] with classical cell signatures in all the other datasets.

### Copy number variation analysis

To investigate the insertion and deletion events in the genomic regions from the TCGA samples, we employed GISTIC 2.0, a revised computational program that identifies somatic copy number alterations from investigation of the amplitude and frequency of observed alteration events [61].

### Differential expression, hierarchical clustering, survival analysis and statistical analysis

Wilcoxon test and Kruskal-Wallis test were used to compare the differences between high- and low-cuproptosis groups regarding multiple features. ‘Limma’ package [62] was used to calculate the differential expression of marker genes between high- and low-cuproptosis groups in pan-cancer. Hierarchical clustering analysis was used to classify the TCGA dataset via the ward.D method with 26 immune-associated genesets as input. In addition, the difference in the proportion of clinical factors between cuproptosis-low and -high groups was computed by Chisq test. The relationships between the score of cuproptosis and immune infiltrating cells were calculated by Pearson’s correlation coefficient. To examine the association between cuproptosis score and survival, Kaplan-Meier survival analysis was performed, and the log-rank test was used to determine the significance of the differences. In pan-cancer survival analysis, patients were equally divided based on their cuproptosis scores to cuproptosis-high and -low groups. In immunotherapy datasets, patients were grouped based on the best cut-off defined by ‘survminer’ R package, with the minprop parameter set to 0.3 to ensure balanced sample distribution and prevent extreme group size disparities. To access the association between cuproptosis score and overall survival, we performed univariate Cox proportional hazard regression. Also, we employed multivariate Cox regression to evaluate the independent prognosis value of cuproptosis score compared with other clinical factors like Tumor Size, T stage, M Stage etc. P or adjP < 0.05 was considered statistically significant.

## Electronic Supplementary Material

Below is the link to the electronic supplementary material.

Supplementary Material 1.

## Data Availability

All data generated or analyzed during this study are included in this article and its supplementary information files. Publicly available datasets were utilized from The Cancer Genome Atlas (TCGA), Gene Expression Omnibus (GEO: GSE91061, GSE133713, GSE157738, GSE22153, GSE98394, GSE100797, GSE79691, GSE67501, GSE103668, GSE19423, GSE72056, GSE115978, GSE137829, GSE139829, GSE123814), dbGaP (phs000452.v3), and the IMvigor210 cohort, with full details provided in Supplementary Table S1.

## References

[1] Waldman AD, Fritz JM, Lenardo MJ. A guide to cancer immunotherapy: from T cell basic science to clinical practice. Nat Rev Immunol. 2020;20(11):651–68. 32433532 10.1038/s41577-020-0306-5 PMC7238960

[2] Murciano-Goroff YR, Warner AB, Wolchok JD. The future of cancer immunotherapy: microenvironment-targeting combinations. Cell Res. 2020;30(6):507–19. 32467593 10.1038/s41422-020-0337-2 PMC7264181

[3] DeBerardinis RJ. Tumor microenvironment, metabolism, and immunotherapy. N Engl J Med. 2020;382(9):869–71. 32101671 10.1056/NEJMcibr1914890

[4] Lei X, et al. Immune cells within the tumor microenvironment: biological functions and roles in cancer immunotherapy. Cancer Lett. 2020;470:126–33. 31730903 10.1016/j.canlet.2019.11.009

[5] Runa F, et al. Tumor microenvironment heterogeneity: challenges and opportunities. Curr Mol biology Rep. 2017;3(4):218–29. 10.1007/s40610-017-0073-7 PMC5802345 29430386

[6] Ge R, Wang Z, Cheng L. Tumor microenvironment heterogeneity an important mediator of prostate cancer progression and therapeutic resistance. NPJ Precision Oncol. 2022;6(1):1–8. 10.1038/s41698-022-00272-w PMC9068628 35508696

[7] Hegde PS, Chen DS. Top 10 challenges in cancer immunotherapy. Immunity. 2020;52(1):17–35. 31940268 10.1016/j.immuni.2019.12.011

[8] Xia L, et al. The cancer metabolic reprogramming and immune response. Mol Cancer. 2021;20(1):1–21. 33546704 10.1186/s12943-021-01316-8 PMC7863491

[9] Peng F, et al. Regulated cell death (RCD) in cancer: key pathways and targeted therapies. Signal Transduct Target Therapy. 2022;7(1):1–66. 10.1038/s41392-022-01110-y PMC9376115 35963853

[10] Tang D, et al. The molecular machinery of regulated cell death. Cell Res. 2019;29(5):347–64. 30948788 10.1038/s41422-019-0164-5 PMC6796845

[11] Galluzzi L et al. Consensus guidelines for the definition, detection and interpretation of immunogenic cell death. J Immunother Cancer, 2020;8(1). 10.1136/jitc-2019-000337 PMC7064135 32209603

[12] Tsvetkov P, et al. Copper induces cell death by targeting lipoylated TCA cycle proteins. Science. 2022;375(6586):1254–61. 35298263 10.1126/science.abf0529 PMC9273333

[13] Ding F, et al. Restoration of the Immunogenicity of Tumor Cells for Enhanced Cancer Therapy via Nanoparticle-Mediated Copper Chaperone Inhibition. Angew Chem. 2022;134(31):e202203546. 10.1002/anie.202203546 35642869

[14] Voli F, et al. Intratumoral copper modulates PD-L1 expression and influences tumor immune evasion. Cancer Res. 2020;80(19):4129–44. 32816860 10.1158/0008-5472.CAN-20-0471

[15] Liu YL, et al. Tetrathiomolybdate (TM)-associated copper depletion influences collagen remodeling and immune response in the pre-metastatic niche of breast cancer. NPJ breast cancer. 2021;7(1):1–11. 34426581 10.1038/s41523-021-00313-w PMC8382701

[16] Wu Q, et al. Metabolic regulation in the immune response to cancer. Cancer Commun. 2021;41(8):661–94. 10.1002/cac2.12182 PMC8360644 34145990

[17] Ma X, et al. CD36-mediated ferroptosis dampens intratumoral CD8 + T cell effector function and impairs their antitumor ability. Cell Metabol. 2021;33(5):1001–12. e5. 10.1016/j.cmet.2021.02.015 PMC8102368 33691090

[18] Drijvers JM, et al. Pharmacologic Screening Identifies Metabolic Vulnerabilities of CD8 + T CellsMetabolic Screening of CD8 + T Cells. Cancer Immunol Res. 2021;9(2):184–99. 33277233 10.1158/2326-6066.CIR-20-0384 PMC7864883

[19] Wiernicki B, et al. Cancer cells dying from ferroptosis impede dendritic cell-mediated anti-tumor immunity. Nat Commun. 2022;13(1):1–15. 35760796 10.1038/s41467-022-31218-2 PMC9237053

[20] Bonati L, Tang L. Cytokine engineering for targeted cancer immunotherapy. Curr Opin Chem Biol. 2021;62:43–52. 33684633 10.1016/j.cbpa.2021.01.007

[21] Kondoh N, Mizuno-Kamiya M. The role of immune modulatory cytokines in the tumor microenvironments of head and neck squamous cell carcinomas. Cancers. 2022;14(12):2884. 35740551 10.3390/cancers14122884 PMC9221278

[22] Wu Y, et al. Revitalizing T cells: breakthroughs and challenges in overcoming T cell exhaustion. Signal Transduct Target therapy. 2026;11(1):2. 10.1038/s41392-025-02327-3 PMC12764927 41484095

[23] Baessler A, Vignali DA. T cell exhaustion. Annu Rev Immunol. 2024;42(1):179–206. 38166256 10.1146/annurev-immunol-090222-110914

[24] Chen DS, Mellman I. Oncology meets immunology: the cancer-immunity cycle. Immunity. 2013;39(1):1–10. 23890059 10.1016/j.immuni.2013.07.012

[25] Kadomoto S, Izumi K, Mizokami A. The CCL20-CCR6 axis in cancer progression. Int J Mol Sci. 2020;21(15):5186. 32707869 10.3390/ijms21155186 PMC7432448

[26] Korbecki J, et al. CC chemokines in a tumor: a review of pro-cancer and anti-cancer properties of the ligands of receptors CCR1, CCR2, CCR3, and CCR4. Int J Mol Sci. 2020;21(21):8412. 33182504 10.3390/ijms21218412 PMC7665155

[27] Ma X, et al. Cholesterol induces CD8 + T cell exhaustion in the tumor microenvironment. Cell Metabol. 2019;30(1):143–56. e5. 10.1016/j.cmet.2019.04.002 PMC7061417 31031094

[28] King RJ, Singh PK, Mehla K. The cholesterol pathway: Impact on immunity and cancer. Trends Immunol. 2022;43(1):78–92. 34942082 10.1016/j.it.2021.11.007 PMC8812650

[29] Chaisaingmongkol J, et al. Common molecular subtypes among Asian hepatocellular carcinoma and cholangiocarcinoma. Cancer Cell. 2017;32(1):57–70. e3. 28648284 10.1016/j.ccell.2017.05.009 PMC5524207

[30] Leone RD, Powell JD. Metabolism of immune cells in cancer. Nat Rev Cancer. 2020;20(9):516–31. 32632251 10.1038/s41568-020-0273-y PMC8041116

[31] Yang RK, et al. Outcome-related signatures identified by whole Transcriptome sequencing of Resectable stage III/IV melanoma evaluated after starting Hu14. 18-IL2. Clin Cancer Res. 2020;26(13):3296–306. 32152202 10.1158/1078-0432.CCR-19-3294 PMC7334053

[32] Maurer DM, et al. Dysregulated NF-κB–Dependent ICOSL Expression in Human Dendritic Cell Vaccines Impairs T-cell Responses in Patients with Melanoma. Cancer Immunol Res. 2020;8(12):1554–67. 33051240 10.1158/2326-6066.CIR-20-0274 PMC8018573

[33] Jönsson G, et al. Gene Expression Profiling–Based Identification of Molecular Subtypes in Stage IV Melanomas with Different Clinical OutcomeMolecular Classification of Stage IV Melanoma. Clin Cancer Res. 2010;16(13):3356–67. 20460471 10.1158/1078-0432.CCR-09-2509

[34] Badal B et al. Transcriptional dissection of melanoma identifies a high-risk subtype underlying TP53 family genes and epigenome deregulation. JCI insight, 2017;2(9). 10.1172/jci.insight.92102 PMC5414564 28469092

[35] Lauss M, et al. Mutational and putative neoantigen load predict clinical benefit of adoptive T cell therapy in melanoma. Nat Commun. 2017;8(1):1–11. 29170503 10.1038/s41467-017-01460-0 PMC5701046

[36] Ascierto ML, et al. Transcriptional Mechanisms of Resistance to Anti–PD-1 TherapyTranscriptional Mechanisms of Resistance to Anti–PD-1. Clin Cancer Res. 2017;23(12):3168–80. 28193624 10.1158/1078-0432.CCR-17-0270 PMC5474192

[37] Ascierto ML, et al. The Intratumoral Balance between Metabolic and Immunologic Gene Expression Is Associated with Anti–PD-1 Response in Patients with Renal Cell CarcinomaTumor-Intrinsic Factors and Anti–PD-1 Resistance in RCC. Cancer Immunol Res. 2016;4(9):726–33. 27491898 10.1158/2326-6066.CIR-16-0072 PMC5584610

[38] Birkbak N, et al. Overexpression of BLM promotes DNA damage and increased sensitivity to platinum salts in triple-negative breast and serous ovarian cancers. Ann Oncol. 2018;29(4):903–9. 29452344 10.1093/annonc/mdy049 PMC5913643

[39] Kim Y-J, et al. Gene signatures for the prediction of response to Bacillus Calmette-Guerin immunotherapy in primary pT1 bladder cancers. Clin Cancer Res. 2010;16(7):2131–7. 20233890 10.1158/1078-0432.CCR-09-3323

[40] Liu D, et al. Integrative molecular and clinical modeling of clinical outcomes to PD1 blockade in patients with metastatic melanoma. Nat Med. 2019;25(12):1916–27. 31792460 10.1038/s41591-019-0654-5 PMC6898788

[41] Van Allen EM, et al. Genomic correlates of response to CTLA-4 blockade in metastatic melanoma. Science. 2015;350(6257):207–11. 26359337 10.1126/science.aad0095 PMC5054517

[42] Necchi A, et al. Atezolizumab in platinum-treated locally advanced or metastatic urothelial carcinoma: post-progression outcomes from the phase II IMvigor210 study. Ann Oncol. 2017;28(12):3044–50. 28950298 10.1093/annonc/mdx518 PMC5834063

[43] Tirosh I, et al. Dissecting the multicellular ecosystem of metastatic melanoma by single-cell RNA-seq. Science. 2016;352(6282):189–96. 27124452 10.1126/science.aad0501 PMC4944528

[44] Jerby-Arnon L, et al. A cancer cell program promotes T cell exclusion and resistance to checkpoint blockade. Cell. 2018;175(4):984–97. e24. 30388455 10.1016/j.cell.2018.09.006 PMC6410377

[45] Dong B, et al. Single-cell analysis supports a luminal-neuroendocrine transdifferentiation in human prostate cancer. Commun biology. 2020;3(1):1–15. 10.1038/s42003-020-01476-1 PMC7745034 33328604

[46] Durante MA, et al. Single-cell analysis reveals new evolutionary complexity in uveal melanoma. Nat Commun. 2020;11(1):1–10. 31980621 10.1038/s41467-019-14256-1 PMC6981133

[47] Yost KE, et al. Clonal replacement of tumor-specific T cells following PD-1 blockade. Nat Med. 2019;25(8):1251–9. 31359002 10.1038/s41591-019-0522-3 PMC6689255

[48] Subramanian A, et al. Gene set enrichment analysis: a knowledge-based approach for interpreting genome-wide expression profiles. Proc Natl Acad Sci. 2005;102(43):15545–50. 16199517 10.1073/pnas.0506580102 PMC1239896

[49] Yu G, et al. clusterProfiler: an R package for comparing biological themes among gene clusters. OMICS. 2012;16(5):284–7. 22455463 10.1089/omi.2011.0118 PMC3339379

[50] Newman AM, et al. Robust enumeration of cell subsets from tissue expression profiles. Nat Methods. 2015;12(5):453–7. 25822800 10.1038/nmeth.3337 PMC4739640

[51] Aran D, Hu Z, Butte AJ. xCell: digitally portraying the tissue cellular heterogeneity landscape. Genome Biol. 2017;18(1):1–14. 29141660 10.1186/s13059-017-1349-1 PMC5688663

[52] Li T, et al. TIMER2. 0 for analysis of tumor-infiltrating immune cells. Nucleic Acids Res. 2020;48(W1):W509–14. 32442275 10.1093/nar/gkaa407 PMC7319575

[53] Becht E, et al. Estimating the population abundance of tissue-infiltrating immune and stromal cell populations using gene expression. Genome Biol. 2016;17(1):1–20. 27908289 10.1186/s13059-016-1113-y PMC5134277

[54] Jiang P, et al. Signatures of T cell dysfunction and exclusion predict cancer immunotherapy response. Nat Med. 2018;24(10):1550–8. 30127393 10.1038/s41591-018-0136-1 PMC6487502

[55] He Y, et al. Classification of triple-negative breast cancers based on Immunogenomic profiling. J Experimental Clin Cancer Res. 2018;37(1):1–13. 10.1186/s13046-018-1002-1 PMC6310928 30594216

[56] Danaher P, et al. Gene expression markers of tumor infiltrating leukocytes. J Immunother Cancer. 2017;5(1):1–15. 28239471 10.1186/s40425-017-0215-8 PMC5319024

[57] Saltz J, et al. Spatial organization and molecular correlation of tumor-infiltrating lymphocytes using deep learning on pathology images. Cell Rep. 2018;23(1):181–93. e7. 29617659 10.1016/j.celrep.2018.03.086 PMC5943714

[58] Yoshihara K, et al. Inferring tumour purity and stromal and immune cell admixture from expression data. Nat Commun. 2013;4(1):1–11. 10.1038/ncomms3612 PMC3826632 24113773

[59] Stuart T, et al. Comprehensive integration of single-cell data. Cell. 2019;177(7):1888–902. e21. 31178118 10.1016/j.cell.2019.05.031 PMC6687398

[60] Aran D, et al. Reference-based analysis of lung single-cell sequencing reveals a transitional profibrotic macrophage. Nat Immunol. 2019;20(2):163–72. 30643263 10.1038/s41590-018-0276-y PMC6340744

[61] Mermel CH, et al. GISTIC2. 0 facilitates sensitive and confident localization of the targets of focal somatic copy-number alteration in human cancers. Genome Biol. 2011;12(4):1–14. 10.1186/gb-2011-12-4-r41 PMC3218867 21527027

[62] Ritchie ME, et al. limma powers differential expression analyses for RNA-sequencing and microarray studies. Nucleic Acids Res. 2015;43(7):e47–47. 25605792 10.1093/nar/gkv007 PMC4402510

