# Supplementary material for: Cuproptosis as a novel predictor of immunotherapy response and shapes the immune landscape in pan-cancer analysis
Source: Discov Oncol. 2026 May 1;17:920. doi: 10.1007/s12672-026-05070-5 (PMC13280067; doi:10.1007/s12672-026-05070-5)
Supplement: Supplementary file 1 — Supplementary Material 1. [file 12672_2026_5070_MOESM1_ESM.doc]

Supplementary data for

**Cuproptosis as a novel predictor of immunotherapy response and shapes the immune landscape in pan-cancer analysis**

Yitong Pan^1,2^, Xiaodi Hu^2#^, Jun Cheng^2#^.

^1^ Furong Laboratory, Central South University, Changsha 410078, Hunan, China.

^2^ Department of Radiation Oncology, Hunan Cancer Hospital and the Affiliated Cancer Hospital of Xiangya School of Medicine, Central South University, Changsha, Hunan, 410013, China.

#Co-corresponding authors:

Jun Cheng

Hunan Cancer Hospital and the Affiliated Cancer Hospital of Xiangya School of Medicine, Central South University, Changsha, Hunan, 410013, China.

Email: chengjun@hnca.org.cn

Xiaodi Hu

Hunan Cancer Hospital and the Affiliated Cancer Hospital of Xiangya School of Medicine, Central South University, Changsha, Hunan, 410013, China.

Email: huxiaodi@hnca.org.cn

**File list:**

Figure S1-5


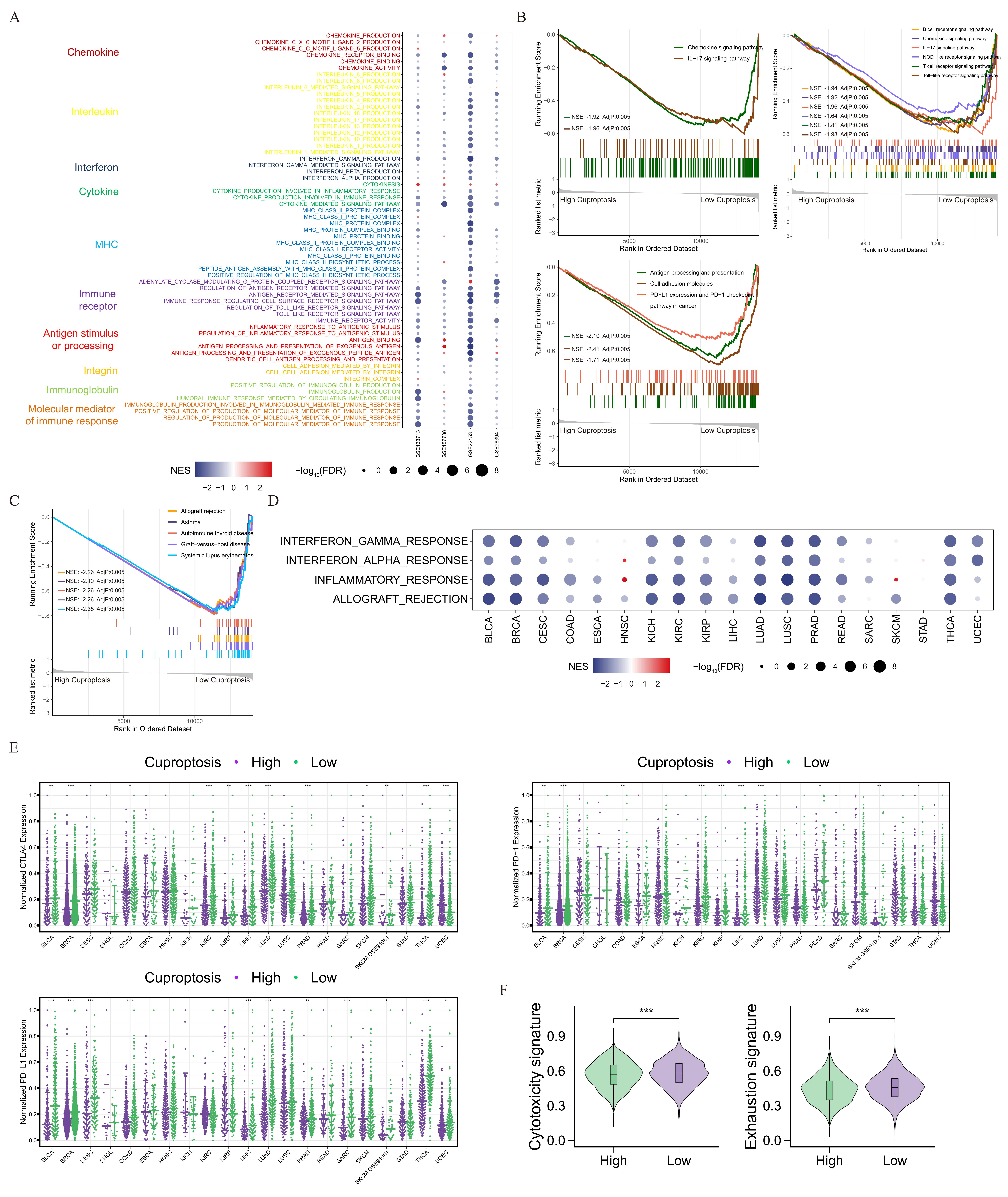


Figure S1. Extrinsic immune landscape: immune function in cuproptosis-low and -high groups. (A) Enrichment of GO pathways associated with immune function in patients with cuproptosis-high and -low groups from TCGA datasets. (B-C) Enrichment of KEGG pathways in patients with cuproptosis-high and -low groups. (D) Enrichment of hallmark pathways in patients with cuproptosis-high and -low groups. (E) Jitter plot of the expression of CTLA4, PD-1 and PD-L1 in patients with cuproptosis-high and -low groups. (F) Violin plot showing GSVA scores of exhaustion and cytotoxicity gene signatures in all integrated TCGA tumor samples. (Wilcoxon test *P < 0.05; **P < 0.01; ***P < 0.001)


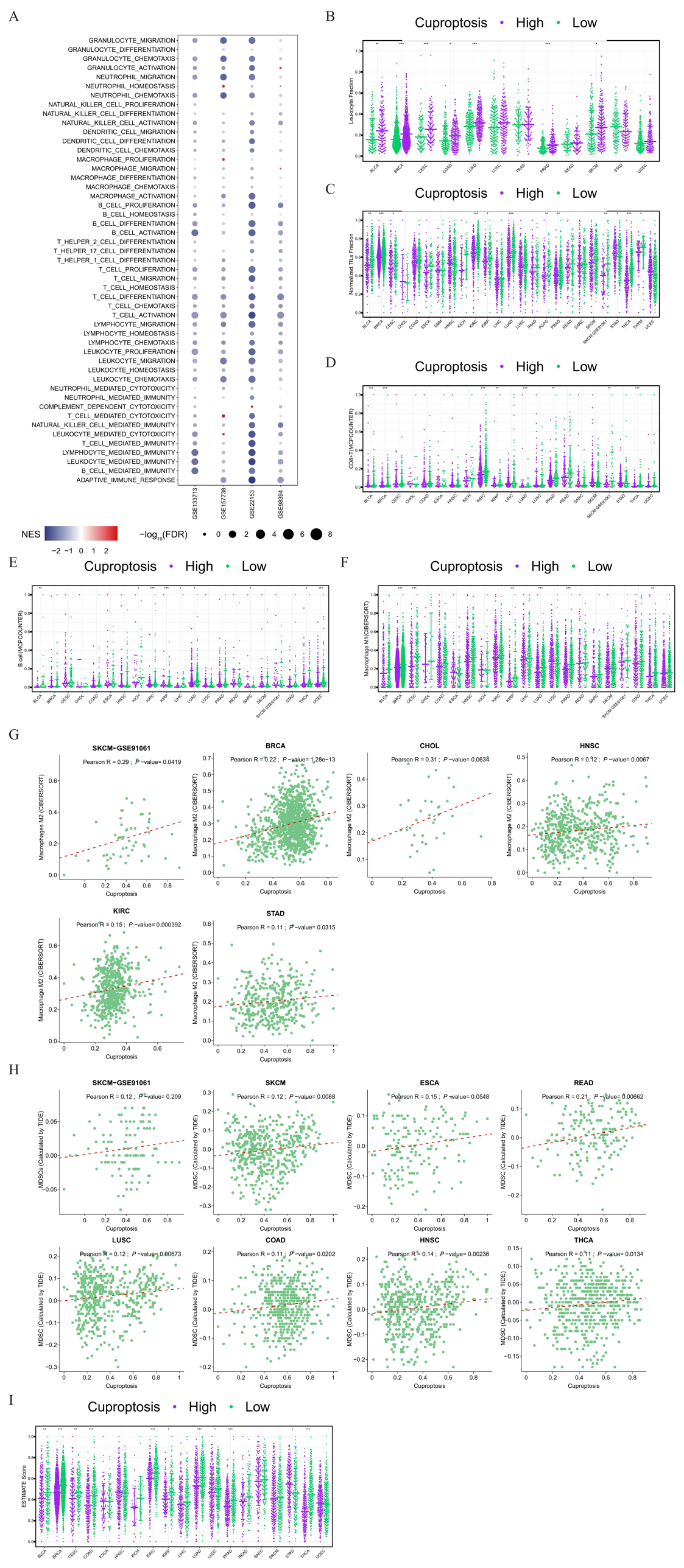


Figure S2. Underlying immune infiltration in the patients with high and low cuproptosis score. (A) Enrichment of GO pathways associated with immune infiltration in SKCM patients with cuproptosis-high and -low groups from GEO datasets. (B-F) Jitter plot of the fraction of leukocytes, TILs, CD8T cells, B cells and macrophage M1 cells in patients with cuproptosis-high and -low groups. (Wilcoxon test ***P < 0.001) (G) The correlation of cuproptosis score and the fraction of macrophage M2 in multiple cancer types. (H) The correlation of cuproptosis score and the fraction of MDSCs calculated by TIDE in multiple cancer types. (D) Jitter plot of the ESTIMATE score calculated by ‘ESTIMATE’ R package in patients with cuproptosis-high and -low groups. (Wilcoxon test *P < 0.05; **P < 0.01; ***P < 0.001)


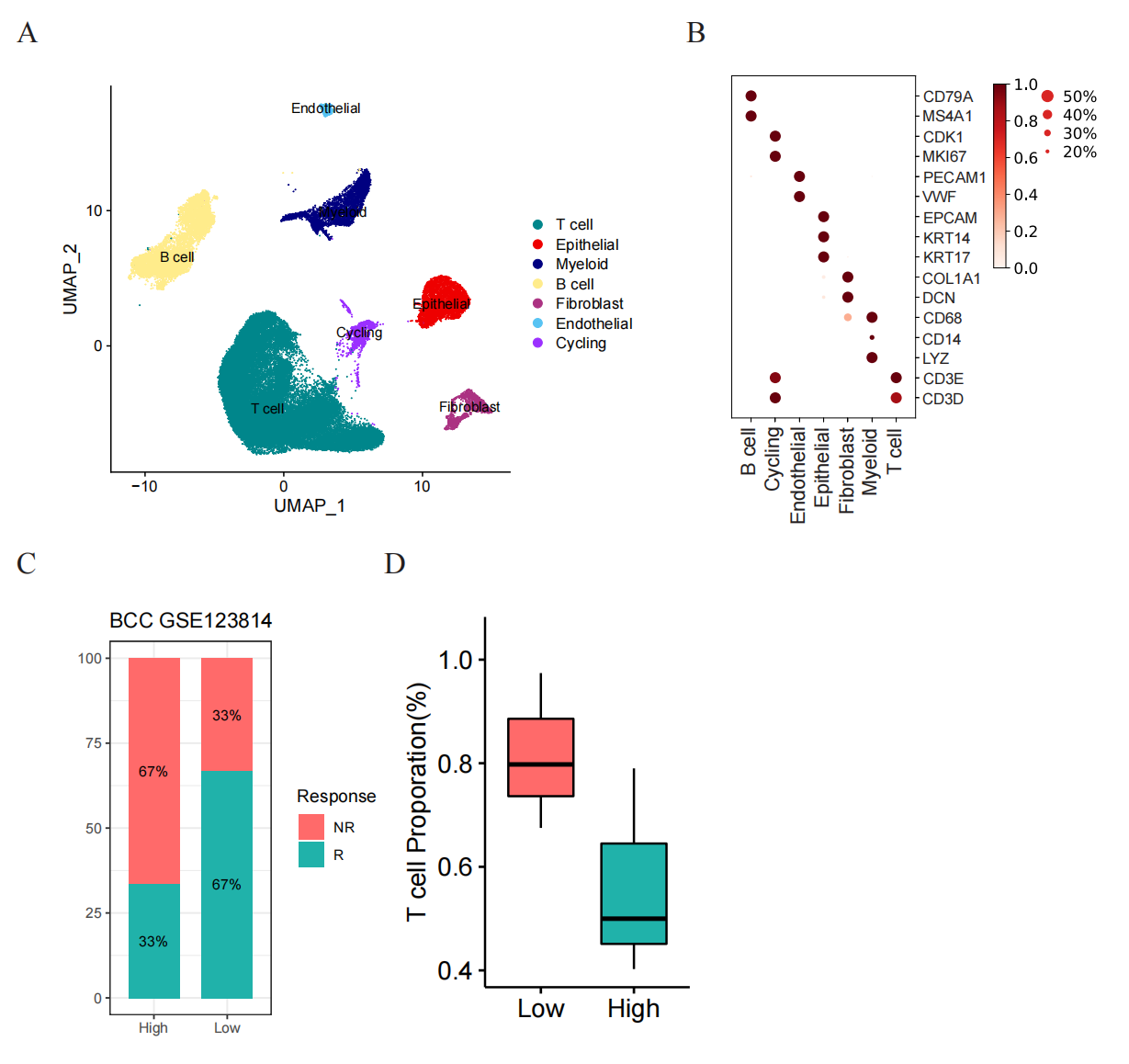


Figure S3. Cuproptosis-related immune landscapes at single-cell resolution upon immunotherapy. (A) UMAP plot showing the composition of 7 main subtypes derived from basal cell carinoma tissues (GSE123814). (B) The expression of markers for 7 cell types. The color of each dots represents the mean expression of marker gene within each cell type and the size of each dots shows the fraction of cells expressing the marker gene in this cell type. (C) The proportion of response status (R: Response, NR: non-response) to immunotherapy in cuproptosis-high and -low groups. (D) Boxplot of the proportion of T cells in cuproptosis -high and -low groups.


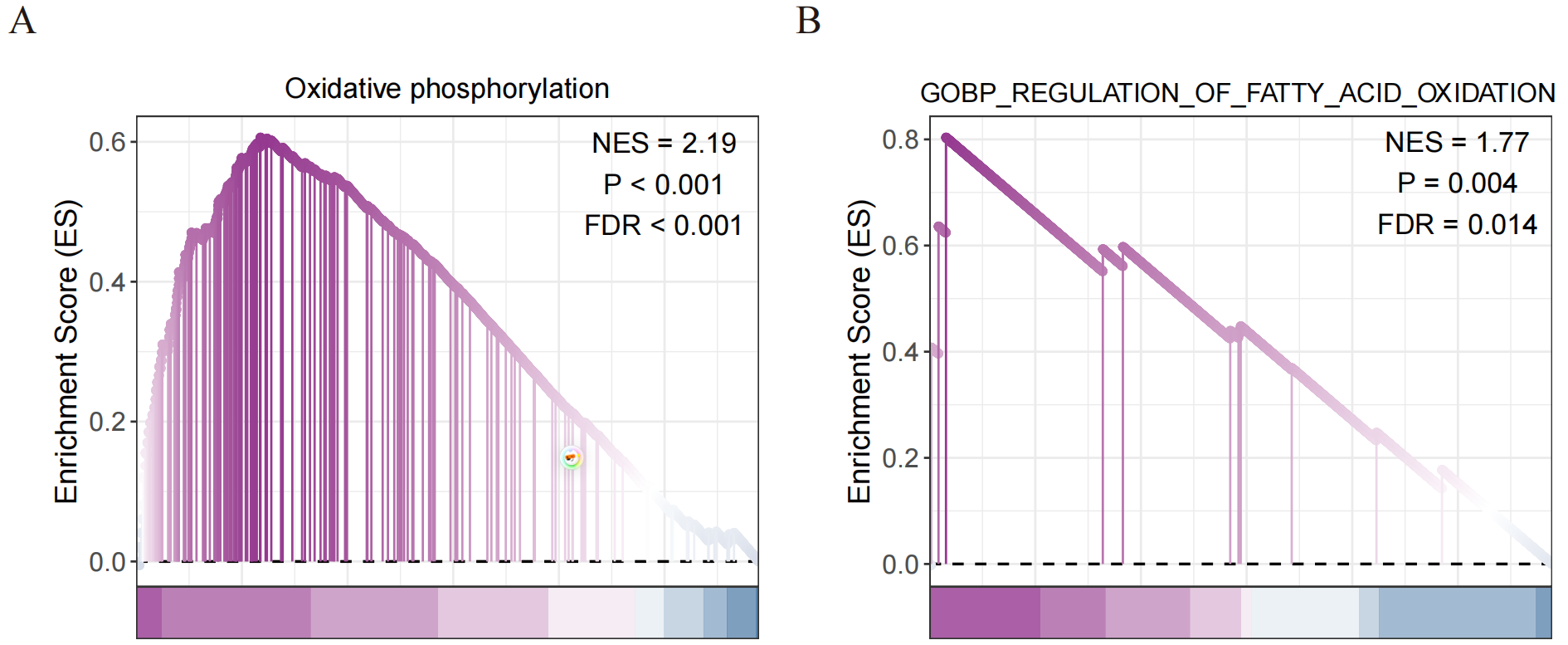


Figure S4. Metabolic characteristics of tumor cells. (A-B) GSEA enrichment profiles of OXPHOS (A) and FAO (B) pathways comparing tumor cells from cuproptosis- high versus -low groups.


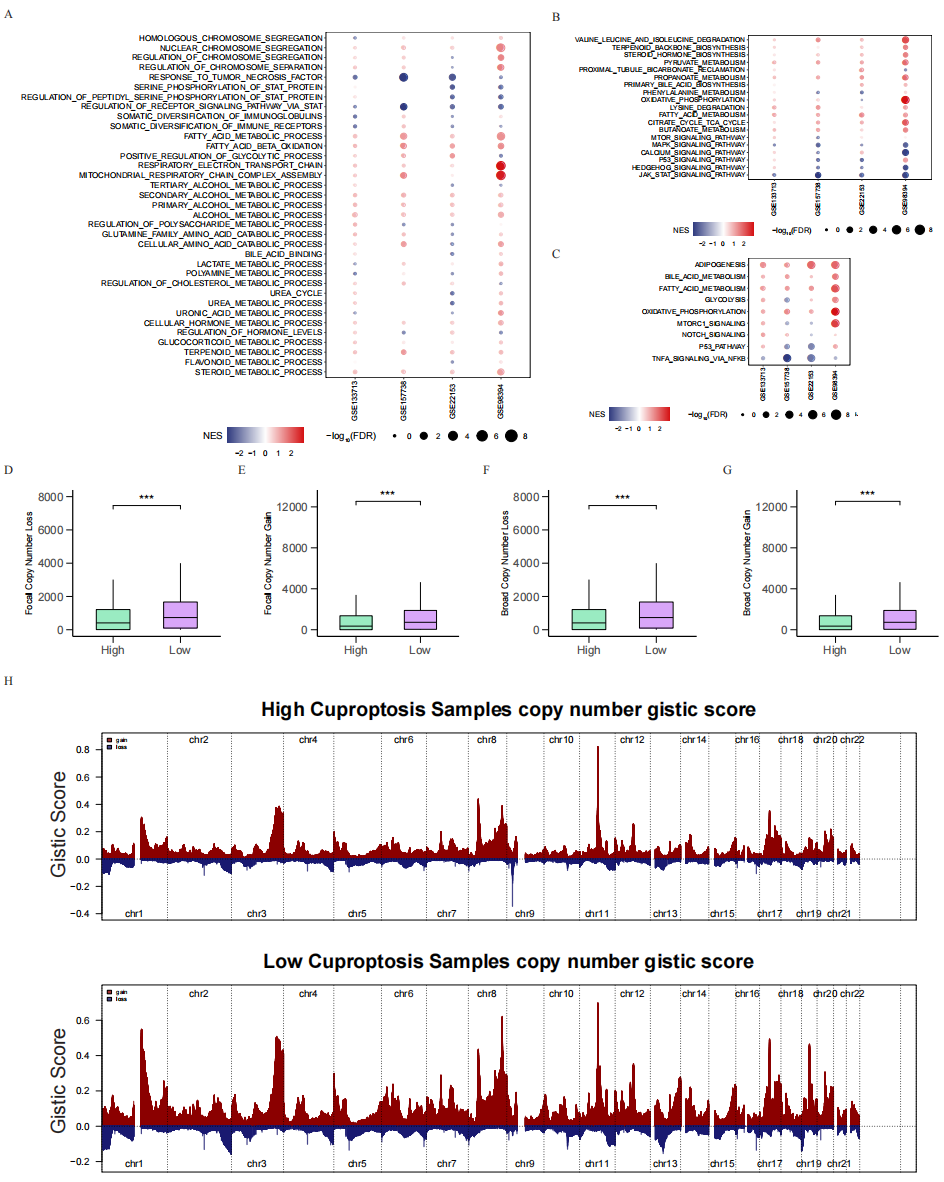


Figure S5. Potential intrinsic immune profiles in the patients with high and low cuproptosis scores. (A-C) Enrichment of pathways associated with immunosuppressive metabolites or metabolic processes in SKCM patients with cuproptosis-high and -low groups from GEO datasets. (D) Boxplot of the loss of focal copy number in cuproptosis-low and -high groups in all-integrated tumor samples. (Wilcoxon test ***P < 0.001) (E) Boxplot of the gain of focal copy number in cuproptosis-low and -high groups in all-integrated tumor samples. (Wilcoxon test ***P < 0.001) (F) Boxplot of the loss of broad copy number in cuproptosis-low and -high groups in all-integrated tumor samples. (Wilcoxon test ***P < 0.001) (G) Boxplot of the gain of broad copy number in cuproptosis-low and -high groups in all-integrated tumor samples. (Wilcoxon test ***P < 0.001) (H-I) The distribution of the amplification or deletion GISTIC score of patients with cuproptosis-low and -high groups.
